# A practical community-based response strategy to interrupt Ebola transmission in sierra Leone, 2014–2015

**DOI:** 10.1186/s40249-016-0167-0

**Published:** 2016-08-05

**Authors:** Zhong-Jie Li, Wen-Xiao Tu, Xiao-Chun Wang, Guo-Qing Shi, Zun-Dong Yin, Hai-Jun Su, Tao Shen, Da-Peng Zhang, Jian-Dong Li, Shan Lv, Chun-Li Cao, Rui-Qian Xie, Hong-Zhou Lu, Rong-Meng Jiang, Zheng Cao, Zhi-Jie An, Lei-Lei Li, Jie Xu, Yan-Wen Xiong, Wei Zang, Wei Zhang, Hong-Wei Zhang, Wen-Sen Chen, Hua Ling, Wen Xu, Jian Cai, Huan-Jin Luo, Xue-Sheng Xing, Can-Jun Zheng, Qiang Wei, Xin-Xu Li, Mei Li, Hai Jiang, Li-Quan Deng, Ming-Quan Chen, Xiang Huo, Feng Xu, Xue-Hui Lai, Xi-Chen Bai, Long-Jie Ye, Jian-Yi Yao, Wen-Wu Yin, Jiao-Jin Sun, Lin Xiao, Fu-Qiang Liu, Xiao-Qiang Liu, Hong-Wei Fan, Zeng-Qiang Kou, Ji-Kun Zhou, Hao Zhang, Da-Xin Ni, Thomas T. Samba, Qun Li, Hong-Jie Yu, Yu Wang, Xiao-Feng Liang

**Affiliations:** 1 Division of Infectious Disease, Key Laboratory of Surveillance and Early-warning on Infectious Disease, Chinese Center for Disease Control and Prevention, Beijing, China; 2 Public Health Emergency Center, Chinese Center for Disease Control and Prevention, Beijing, China; 3 National Center for AIDS/STD Control and Prevention, Chinese Center for Disease Control and Prevention, Beijing, China; 4 Chinese Field Epidemiology Training Program, Chinese Center for Disease Control and Prevention, Beijing, China; 5 National Immunization Program, Chinese Center for Disease Control and Prevention, Beijing, China; 6 Bureau of Disease Prevention and Control, National Health and Family Planning Commission of the People’s Republic of China, Beijing, China; 7 National Institute for Viral Disease Control and Prevention, Chinese Center for Disease Control and Prevention, Beijing, China; 8 National Institute of Parasitic Disease, Chinese Center for Disease Control and Prevention, Shanghai, China; 9 Chinese Center for Health Education, Beijing, China; 10 Department of Infectious Diseases, Shanghai Public Health Clinical Center, Shanghai, China; 11 Beijing Ditan Hospital, Capital Medical University, Beijing, China; 12 Health News, Beijing, China; 13 National Institute for Communicable Disease Control and Prevention, Chinese Center for Disease Control and Prevention, Beijing, China; 14 Department of Training, Chinese Center for Health Education, Beijing, China; 15 Center for Infectious Diseases, Beijing Youan Hospital, Capital Medical University, Beijing, China; 16 Infection Management, The First Affiliated Hospital of Nanjing Medical University, Nanjing, China; 17 Chongqing Municipal Center for Disease Control and Prevention, Chongqing, China; 18 Yunnan Provincial Center for Disease Control and Prevention, Kunming, China; 19 Division of Infectious Disease Control and Prevention, Zhejiang Provincial Center for Disease Control and Prevention, Hangzhou, China; 20 Guangdong Provincial Center for Disease Control and Prevention, Guangzhou, China; 21 Division of Acute Infectious Disease Control and Prevention, Hubei Provincial Center for Disease Control and Prevention, Wuhan, China; 22 Office of laboratory Management, Chinese Center for Disease Control and Prevention, Beijing, China; 23 National Center for Tuberculosis Control and Prevention, Chinese Center for Disease Control and Prevention, Beijing, China; 24 Jilin Provincial Center for Disease Control and Prevention, Changchun, China; 25 Department of Infectious Diseases, Huashan Hospital Affiliated to Fudan University, Shanghai, China; 26 Department of Acute Infectious Disease, Jiangsu Provincial Center for Disease Control and Prevention, Nanjing, China; 27 Department of Infectious Diseases, The Second Affiliated Hospital of Zhejiang University School of Medicine, Hangzhou, China; 28 Zhongshan Center for Disease Control and Prevention, Zhongshan, Guangdong Province China; 29 China Population Communication Center, Beijing, China; 30 Jingzhou Center for Disease Control and Prevention, Jingzhou, Hubei Province China; 31 Hunan Provincial Center for Disease Control and Prevention, Changsha, China; 32 Peking Union Medical College Hospital, Beijing, China; 33 Shandong Provincial Center for Disease Control and Prevention, Jinan, China; 34 Shijiazhuang Center for Disease Control and Prevention, Shijiazhuang, Hebei Province China; 35 District Health Management Team, Western Area, Sierra Leone; 36 Chinese Center for Disease Control and Prevention, Beijing, People’s Republic of China

**Keywords:** Ebola virus disease, Community engagement, Health education, Outbreak control

## Abstract

**Background:**

The Ebola virus disease spread rapidly in West Africa in 2014, leading to the loss of thousands of lives. Community engagement was one of the key strategies to interrupt Ebola transmission, and practical community level measures needed to be explored in the field and tailored to the specific context of communities.

**Methods:**

First, community-level education on Ebola virus disease (EVD) prevention was launched for the community’s social mobilizers in six districts in Sierra Leone beginning in November 2014. Then, from January to May of 2015, in three pilot communities, local trained community members were organized to engage in implementation of EVD prevention and transmission interruption measures, by involving them in alert case report, contact tracing, and social mobilization. The epidemiological indicators of transmission interruption in three study communities were evaluated.

**Results:**

A total of 6 016 community social mobilizers from 185 wards were trained by holding 279 workshops in the six districts, and EVD message reached an estimated 631 680 residents. In three pilot communities, 72 EVD alert cases were reported, with 70.8 % of them detected by trained local community members, and 14 EVD cases were finally identified. Contact tracing detected 64.3 % of EVD cases. The median duration of community infectivity for the cases was 1 day. The secondary attack rate was 4.2 %, and no third generation of infection was triggered. No health worker was infected, and no unsafe burial and noncompliance to EVD control measures were recorded. The community-based measures were modeled to reduce 77 EVD cases, and the EVD-free goal was achieved four months earlier in study communities than whole country of Sierra Leone.

**Conclusions:**

The community-based strategy of social mobilization and community engagement was effective in case detection and reducing the extent of Ebola transmission in a country with weak health system. The successfully practical experience to reduce the risk of Ebola transmission in the community with poor resources would potentially be helpful for the global community to fight against the EVD and the other diseases in the future.

**Electronic supplementary material:**

The online version of this article (doi:10.1186/s40249-016-0167-0) contains supplementary material, which is available to authorized users.

## Multilingual abstracts

Please see Additional file 1 for translation of the abstract into six official working languages of the United Nations.

## Background

The Ebola virus disease (EVD) is one of the most serious viral diseases currently known, with a high case-fatality rate around 50 % (20–90 %), and there is no specific treatment and no licensed Ebola vaccines [1]. The EVD outbreak in the western African countries in 2014, in all its unprecedented dimensions, severity and complexity, has become an emergency of international concern and a global public health crisis [2, 3]. The country of Sierra Leone was severely impacted by the outbreak, experiencing 14 122 EVD cases and 3 955 deaths as of 7 November 2015, when World Health Organization (WHO) declared that Ebola virus transmission had been stopped in Sierra Leone [4]. In Sierra Leone, the proportion of literacy among people aged 15 and above was 44 % in 2012, and there were only 0.02 physicians per 1 000 people in 2010 [5]. Lack of knowledge about disease transmission and a weak public health infrastructure contributed to the spread in this country [5–7].

The Ebola virus is transmitted to people from wild animals and spreads in the human population through human-to-human transmission via direct contact with the blood, secretions, organs or other bodily fluids of infected people, and with surfaces and materials contaminated with these fluids [8]. The EVD epidemic in 2014 was marked by intense urban transmission, widely spreading in the community, and multiple outbreaks in health care facilities [9]. To interrupt the Ebola transmission chain between persons, timely case detection and rapid isolation of infected persons is necessary [10, 11]. In Sierra Leone, much of the general technical guidance on response to EVD was developed and implemented under the guidance of WHO [12, 13]. However, the operational implementation to interrupt disease transmission at the community level needed to be explored in the field and tailored to the specific context of communities in Sierra Leone to ensure that the response measures were performed thoroughly and effectively.

Beginning in November 2014, the Chinese public health experts in Sierra Leone, in cooperation with local partners, launched a comprehensive community-based response strategy to interrupt Ebola transmission in the community. This report describes the implementation of this strategy and its impact on Ebola transmission interruption in the pilot communities.

## Methods

The community-based response strategy in Sierra Leone consisted of two parts (see Additional file 2: Appendix File S1): the first was to conduct widespread community education on EVD prevention at the community level in the six districts; And the second one was to carry out field-operational intensified control measures at community level to interrupt the Ebola transmission in three pilot communities, by involving the local community members to participate in the implementation of EVD surveillance and response action in their respective settings.

### Widespread community education in six districts

In Sierra Leone, there are a total of 14 districts and 394 wards (the smallest administrational level), with a population of about 6 million nationwide. As required by the Ministry of Health and Sanitation of Sierra Leone (MOHS-SL), the 6 districts most seriously affected by EVD were selected to perform community education for community social mobilizers. These districts included Western Area Urban, Western Area Rural, Port Loko, Bombali, Tonkolili and Moyamba, which have a total population of nearly 3.5 million. People involved in social mobilization in these communities, including community and religious leaders, community activists, primary health-care workers, and volunteers, were selected to be trained. The workshop mainly included messages on EVD, infection prevention in the community, and skills needed for social mobilization, which are in accordance with the EVD health messages from WHO [7]. Trainees were asked to promise to distribute the messages of EVD prevention to their community members via face-to-face communicating, or distribute posters and brochures, and they were provided with a health package containing some posters, brochures, one thermometer, soap, hand sanitizer and T-shirt with EVD message. Some of the trainees were sampled conveniently to survey their understanding of the EVD messages and their follow-up social mobilization activities in their communities.

### Intensified field measures in three pilot communities

#### Pilot sites selection

From January 13 to May 19, 2015, an intensified field operational EVD response program was performed in three pilot communities with high risk of Ebola transmission. The Jui, Kossoh town, and Grafton communities in the Western Area Rural District which are located in the south-eastern part of the capital city, Freetown, were selected as the field sites, as they are near one of the EVD treatment center and one Ebola testing laboratory. These three communities have a combined area of about 10 km^2^, with about 9000 households and 40 thousands of inhabitants.

#### Community response team recruitment and deployment

The community and religious leaders and activists in the pilot communities who had a high school or higher education level or had some health educational background were recruited and trained to form the local community response team. All the team members came from the three pilot communities, who are familiar with the persons and environment of the community. For the three communities combined, this response team included 60 social mobilizers, 5 disease surveillance officers (DSO), 18 contact tracers, and 3 support staff. All the field work in the three pilot communities was supervised by 5 experienced senior supervisors and 5 field supervisors from the Western Area District Health Management Team. In addition, 2 senior coordinators and 3 community coordinators were enrolled to facilitate the EVD response from the community level to the district and national levels. All the recruited community team members were systematically trained on their roles and how to implement their task in the community.

A “sector” approach, which divided the three pilot communities into a total of 30 subsections with on average 1.3 thousand of population for each subsection, was taken to ensure that all the community households and residents were fully covered by the community response team. The recruited social mobilization, contact tracers, DSO, and field supervising were sub-grouped to be fixed on the 30 corresponding designated subsections (Additional file 2: Appendix File S2 Table S2_1).

#### Operational response mechanism in the community

**Fig. 1:**
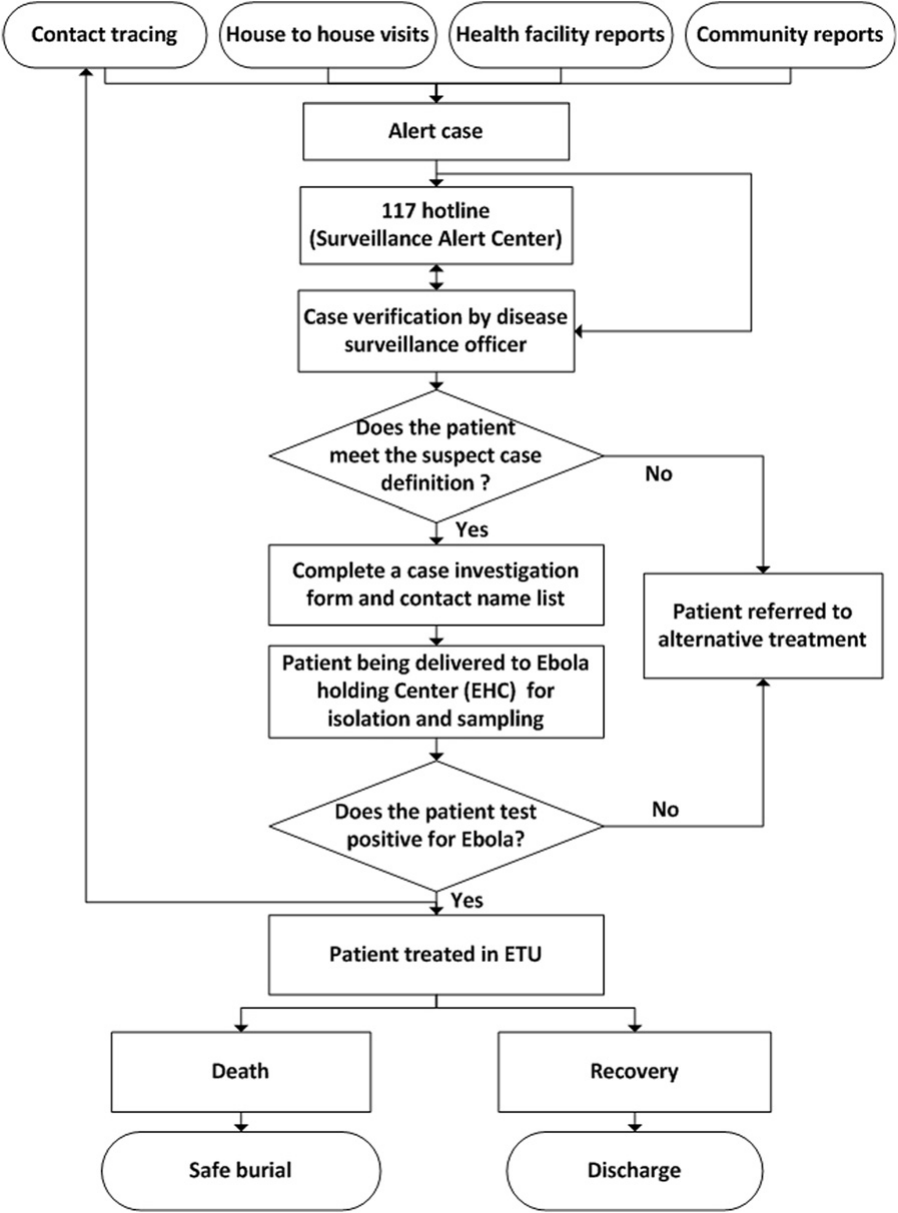
Field-operational workflow of EVD case detection, investigation and management in three pilot communities, Sierra Leone

In accordance with the national guidelines issued by WHO and MOHS-SL, [3] the field-operational proposal on intensified surveillance and response of EVD was further tailored for the community level, and the corresponding EVD case definitions on alert case, suspected case, probable case, confirmed case, non-case and case contact were determined (see Additional file 2: Appendix File S2). The critical components of the response to Ebola transmission in the community, including case detection, verification and investigation, contact tracing and case isolation, are demonstrated in the operational workflow figure (Fig. 1).

Four active detection routes on EVD alert cases were established in the community, including contact tracing, house-to-house visits, health facility reports, and community reports. Each EVD alert case would be promptly investigated by the DSO. Once verified as a suspected case, the case would then be isolated and treated in the Ebola holding center (EHC). Furthermore, if the case’s biological specimen was positive for Ebola virus, this confirmed case would then be treated in the Ebola Treating Center (ETC).

Once a suspected EVD case was identified, any person who met the definition of being a contact to this suspected case would be identified through contact tracing and registered as a contact. Once the suspected case was identified as a probable or confirmed EVD case, the listed contact persons were monitored daily on health status by the designated community contact tracer team for 21 consecutive days after their last contact with the EVD case.

Social mobilization and community engagement were advocated in the community. Each social mobilization team was required to conduct daily house-to-house screening and to distribute posters and leaflets containing key EVD messages to the households in their communities, and to observe the community compliance to the response measures. In addition, billboards and banners on EVD prevention and case reporting were set up in the community.

#### Data collection

The data on investigation of EVD cases and contacts were recorded into the relevant forms by a community response team, and each of which was then required to submit the data to the community coordinator on a daily basis. The key epidemiological indicators of Ebola transmission interruption in the whole country of Sierra Leone including proportion of new confirmed cases from registered contacts, community infectivity time, proportion of confirmed EVD died in the community, unsafe burials for probable or confirmed EVD case, and community compliance were obtained from the weekly situation report by WHO [3].

#### Data analysis

The case fatality rate was calculated as the percentage of fatal EVD cases among the probable or confirmed cases with a known definitive clinical outcome. The interval from symptom onset to hospitalization in EHC or ETU was considered to be community infectivity time. The secondary EVD case denoted the person who was diagnosed as EVD, after having contact with the primary EVD case within the incubation period. Secondary attack rate was calculated by taking the number of secondary EVD cases among the contacts divided by the number of contact persons.

The key epidemiological indicators of Ebola transmission interruption were compared between the three pilot communities and the whole country of Sierra Leone, from January 13 to April 5, 2015. The duration and size of the EVD outbreak in the three pilot communities where the intensified response strategy was implemented were compared with simulated duration and size for the same geographic area under the assumption of no intensified response strategy being implemented. This was done by applying the EVD Response modeling tool developed by United States Center for Disease Control and Prevention, which is a susceptible, incubation, infectious and recovery (SIIR) Model to estimate the number of EVD cases in a community [14]. The details on the methods and parameters of the simulation model are presented in the Additional file 2: Appendix File S3.

## Results

### Outcome of widespread community-based training

**Fig. 2:**
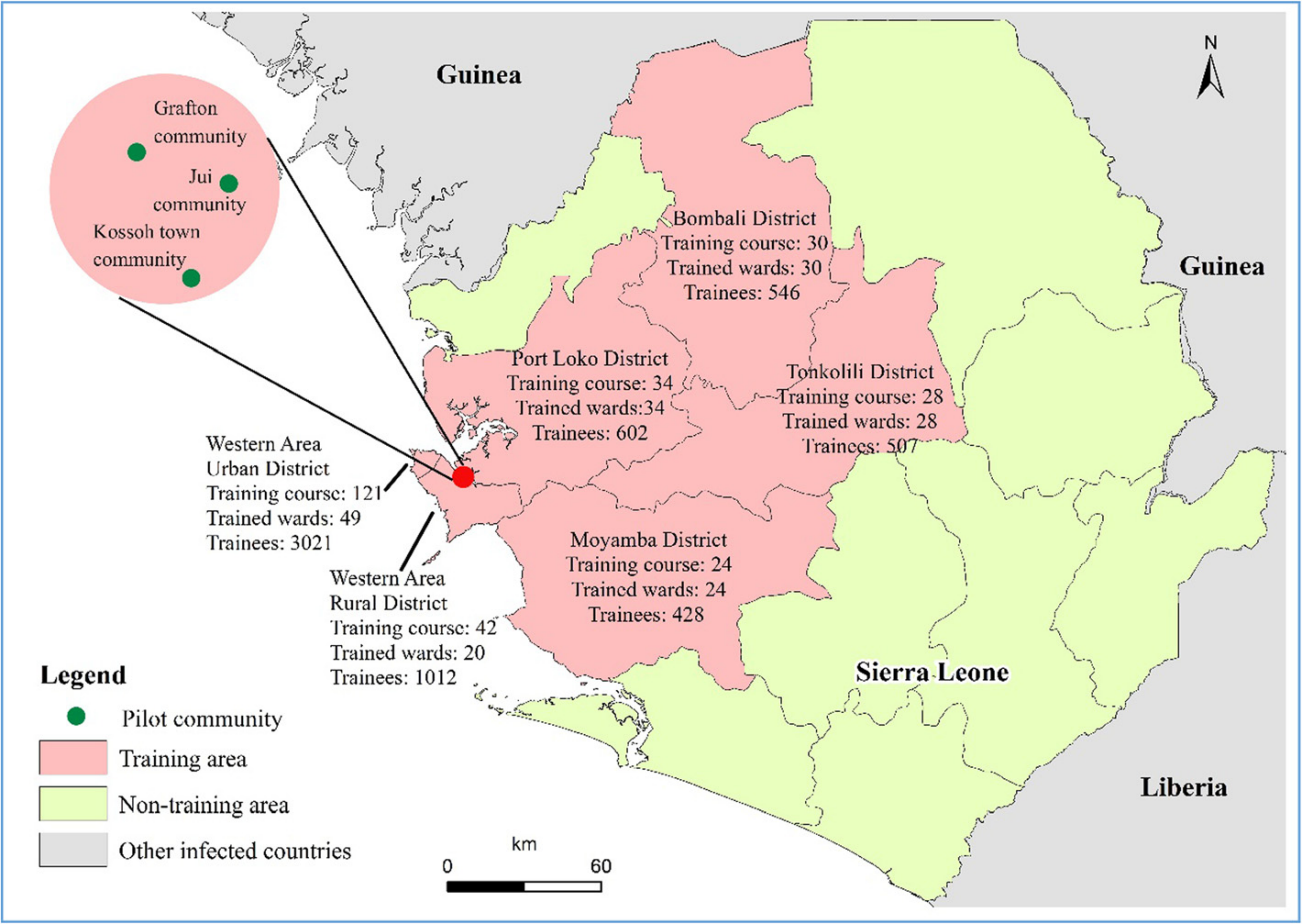
The geographic distribution of community education in six districts and the field intensified response action in three pilot communities against EVD in Sierra Leone, from November 9^th^, 2014 to May 19^th^, 2015

From November 13, 2014 to February 5, 2015, 42 Chinese public health experts in Sierra Leone held 279 workshops and trained 6 016 community social mobilizers from all 185 wards in the targeted six districts, which accounted for 47.0 % of all the 394 wards nationwide (Fig. 2). Among the 207 participants in this workshop that were surveyed, 96 % of them were able to demonstrate complete understanding of the message of EVD prevention in the community. Among 380 trainees surveyed by telephone one month after being trained, 51.1 % had actively participated in social mobilization and health education activities, and on average, each trainee disseminated the EVD messages to 105 other community members. As a result, an estimated 631 680 community members received EVD prevention messages in the community from the 6 016 trained social mobilizers.

### EVD case detection and contact tracing in the community

**Fig. 3:**
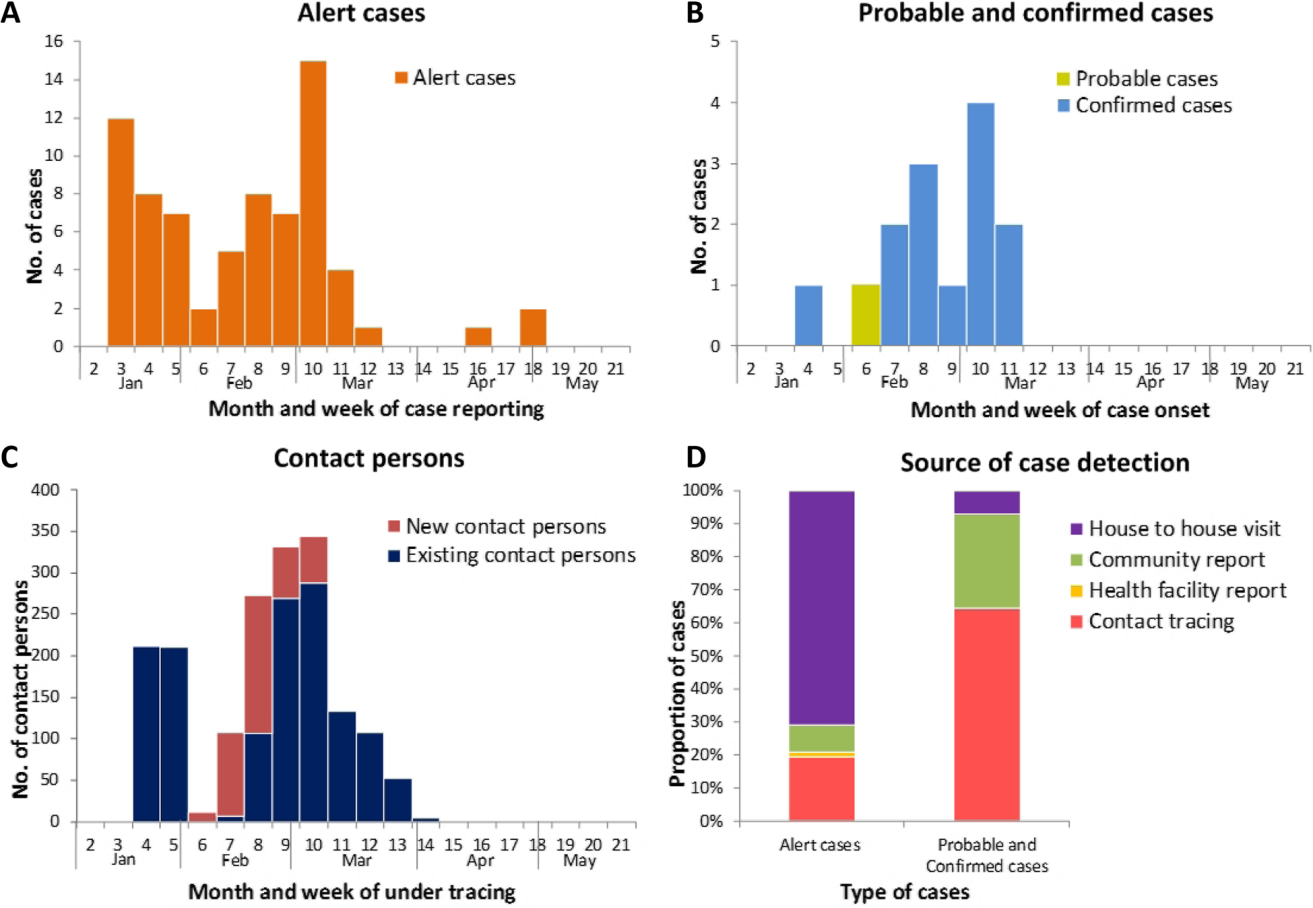
Time series of EVD alert cases, probable and confirmed cases, and contact persons by week and the initial detection source of alert cases and probable and confirmed cases from January 13 to May 19, 2015, in three communities, Sierra Leone. (**a** EVD alert cases; **b** EVD probable and confirmed cases; **c** EVD contact persons under tracing; **d** the initial detection source of EVD alert, probable and confirmed cases.)

During the intensified response period from January 13 to May 19, 2015 in three communities, a total of 72 EVD alert cases were reported, and the last alert case was reported on May 2, 2015 (Fig. 3a); Among these alert cases, 14 EVD cases, including 1 probable and 13 confirmed EVD cases, were further identified, with the final one emerging on March 14 (Fig. 3b). A cumulative total of 607 contacts had been registered and placed under quarantine during the pilot period, and the last contact person finished the 21-day follow up on April 6 (Fig. 3c). House to house visits by social mobilization team generated the largest number of alert cases, accounting for 70.8 % (51/72) of all alert cases, but only one alert case detected from this source was finally identified as a confirmed case. Contact tracing identified 14 alert cases, which accounted for 19 % (14/72) of the total alert cases; of these, 9 were confirmed EVD cases identified during this period. Community reports triggered 6 alert cases, and detected three confirmed cases and one probable case. Health facilities only reported one alert case, which was negative for Ebola (Fig. 3d).

### Community transmission of EVD cases

Among the 14 EVD cases, the median age was 26.5 years (range, 3–55 years), and 6 (46 %) were male. The major occupations of EVD cases included petty trader (4 cases) and student (3 cases), with no EVD case identified among the health care workers. The most common symptoms among the 12 cases with clinical information collected, included fever (83 %), vomiting or nausea (75 %), anorexia (58 %), conjunctivitis (50 %), muscle pain (50 %), joint pain (50 %), and only 1 case (8 %) presented with unexplained bleeding. The median interval from last contact with the EVD cases to onset of illness was 8 days (range, 4–18 days). Among the 8 death EVD cases (case fatality rate of 57.1 %), 7 died in an ETU and 1 case died at home; All were buried in a safe manner. The median interval from onset of illness to death was 4 days (range, 1–7).

**Fig. 4:**
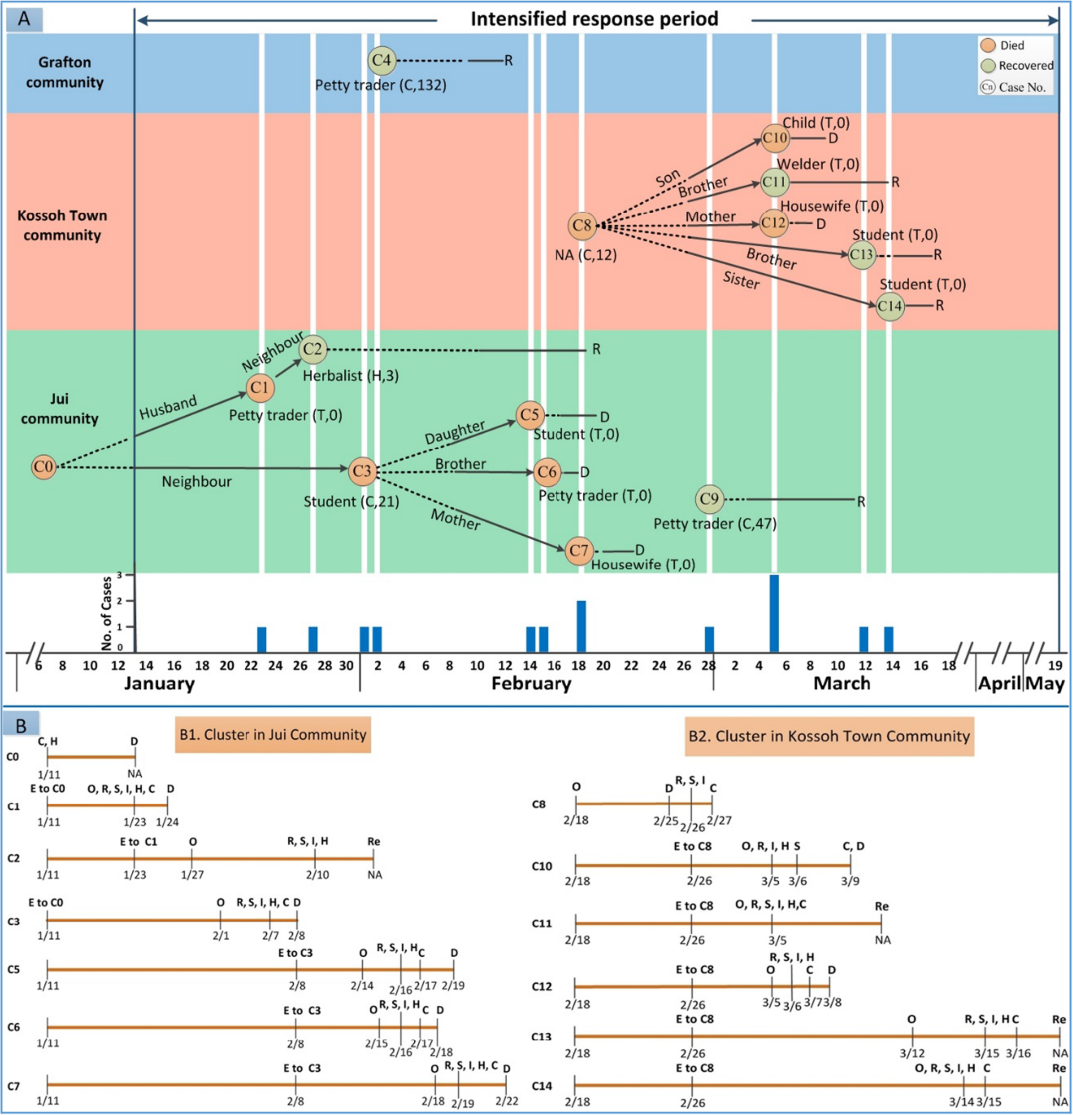
Transmission tree of EVD cases and the timeline of two community clusters in Jui, Kossoh town, and Grafton community, Sierra Leone, from January 13 to May 19, 2015. Panel A: Transmission tree for the EVD cases in three communities of Sierra Leone, January 13-May 19, 2015. In this transmission tree diagram, EVD cases are identified by sequential numbers in each small circle based on date of onset, and labeled with the occupation (source of case detection, amount of contact persons for each case). NA-not available for occupation of C8. On the abbreviation of the source of case detection: T-contact tracing, C-community report, H-house to house visit. Each EVD cases (small circle) is followed by a period of community infectivity time (dotted line); time from date of hospitalization in ETU isolation or safe burial to onset of the next generation case (black arrow); and time from date of hospitalization in ETU or safe burial to final outcome (solid black line), D-died, R-recovered. Panel B: Timeline of exposure history, clinical and control procedure for two cluster of EVD cases in Jui community (Panel B1) and in Kossoh Town community (Panel B2). The abbreviation: E-last exposure to probable or confirmed EVD cases; O-onset of illness; R-case report; S-specimen collection; I-case investigation; H-hospitalization in ETU; C-Being identified as probable or confirmed EVD case; D-died; Re-recovery; NA-not available

Among the 14 EVD cases, 7 cases were detected in Jui community, 6 cases reported in Kossoh Town community, with only 1 case in Grafton community (Fig. 4). Three cases (C4, C8 and C9) were imported cases infected with EVD outside of their local community, and the other 11 cases were infected in their local community. Detailed information on epidemiological relationship is provided in Additional file 2: Appendix File S4.

Two clusters of EVD cases were identified, with the one cluster (6 cases) occurring in Jui community, and the other one (6 cases) in Kossoh Town community (Fig. 4). Among the 215 contact persons, 9 secondary cases were identified, with an overall secondary attack rate of 4 · 2 %; no third generation of case was generated. All nine secondary cases had a history of exposure to an infected person who died, either on the date of death or one day prior to death. In contrast, among all the 170 contacts exposed to the two alive cases (C4 and C9), no one EVD case was detected. Among the 9 secondary EVD cases, 8 cases were the family member of the infector and were identified from the contact persons being monitored; and only 1 case (C2, neighbor of the infector) was reported by local health team via house to house visit, who visited C1 secretly and was not recorded on the contacts list.

#### Evaluation on transmission interruption in the community

The proportion of new confirmed cases detected from the registered contacts in the three communities (64.3 %) was higher than the proportion nationwide (45.6 %) (Table 1). The community infectivity time in three communities was 1.0 day (range, 0–14 days), which is shorter than that of nationwide (1.9 days). The proportion of confirmed EVD deaths in the community was 12.5 % (1/8), which is much less than the overall national statistics (21.2 %). No unsafe burial or noncompliance with EVD control measures were observed by the community response team in the three pilot communities.

**Table 1:** Comparison of epidemiological indicators of transmission interruption between the three pilot communities and the whole country of Sierra Leone, from January 13 to April 5, 2015^a^

Indicators	Nationwide^b^	Three pilot communities
Proportion of new confirmed cases from registered contacts % (no. new confirmed cases/all registered contacts)	45.6 (302/662)	64.3 (9/14)
The median of community infectivity time (range)-days	1.9	1.0 (0–14)
Proportion of confirmed EVD died in the community % (no. EVD cases died in the community/all EVD death cases)	21.2 (156/736)	12.5 (1/8)
Amount of unsafe burials for probable or confirmed EVD case	173	0
Number of districts with at least one security incident or other form of incompliance to EVD control measure (no. per week)	2.7	0

^a^As the last case in the three communities was isolated on March 15, 2015, and the longest incubation period of EVD was 21 days, we set the end date of comparison period to April 5, 2015

^b^All the nationwide indicators of EVD control performance for Sierra Leone were obtained and calculated from WHO situation reports. EVD: Ebola Virus Disease

**Fig. 5:**
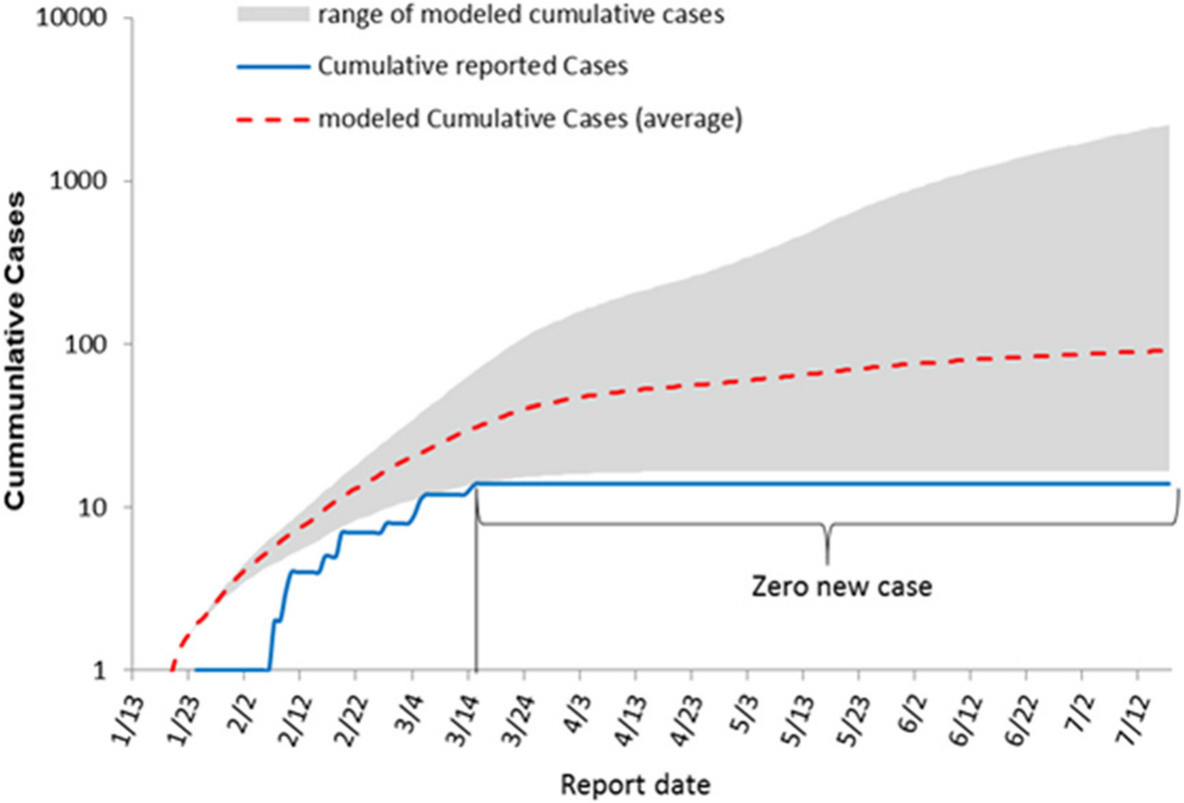
Modeling the number of EVD cases among the three communities, Western Area Rural District, Sierra Leone, from January 13, 2015 to July 17, 2015

Assuming that the key epidemiological indicators of transmission interruption in the three pilot communities were the same as those nationwide during the period of January 13, 2015 to July 17, 2015, it is estimated by the model that there would have been 91 (range from 17 to 2 205) EVD cases occurring in the pilot communities, which was 77 cases more than what was observed (14 cases). Under these assumptions, the zero-EVD goal would not have been achieved until July 17, 2015, a delay of more than 4 months from what was observed in the pilot communities (Fig. 5).

## Discussion

In this study, we found that community-based education for the local residents with face to face communication, especially for the influential community persons was an effective means of widely disseminating EVD prevention messages to members of EVD-affected communities. By conducting intensified response measures in the three pilot communities, EVD alert cases were detected in a timely manner, and the community infectivity time of EVD cases was shortened. All the key epidemiological indicators of transmission interruption in the pilot communities were better than that calculated nationwide in Sierra Leone. The enhanced measures were estimated to reduce the expected number of EVD cases by 77, and the EVD-free goal was achieved much earlier in the pilot community.

In the West African EVD epidemic, in absence of an effective EVD vaccine, community-based risk reduction measures were among the best ways to interrupt Ebola transmission and can be effective even in areas with weak health infrastructure [15, 16]. It is reported in several studies that community education and social mobilization could facilitate public awareness and improve the compliance of community members with prevention and control measures in their communities [15–17]. In our experience, the means of widespread community education should be tailored to the context of the community level. Firstly, a training of trainer model was proposed to train the 40 local trainers by Chinese public health experts in English language, and then the local trainers began to train the community members face to face with simple words and local language, which ensure that education messages was easily understood by the participants from the community. Secondly, we invited the district medical officer to coordinate and enroll the participants via the local community network, and also provided the transportation fees and one free lunch meal for each participant, which propagated the workshop messages to reach the marginal groups in the remote rural areas. Furthermore, some of the posters, leaflets, and banners with simple and understandable key messages on EVD prevention were distributed to each trainee, and they used them to communicate the EVD information in their communities.

In Sierra Leone, the lack of well-trained, professional public health staff presented a great challenge to the goal of implementing Ebola virus control measures [5, 7]. In our experience in the three pilot communities, we used the national response strategy and technical guidance established by WHO and other organizations and helped adapt it to fit the local context by training and supporting the community response teams, who were then the most motivated and skilled at applying it in their own communities. Our practice demonstrated that local community members, with proper educational background, could be recruited and trained to effectively conduct the intensified measure of case detection, investigation, reporting, and contact tracing in their local community under the supervision of experienced professionals from the local public health institute, and contribute to the final achievement of zero-EVD goal. This approach has also been successfully practiced in certain rural areas of Liberia by WHO and US CDC [18, 19]. The way of motivating the community members to engage in EVD control measures in their community provided a feasible solution to overcome the challenges of lack of public health human resources in the community.

This study revealed features of the occurrence and transmission of EVD cases in the community which should be addressed to reach the EVD-free goal. First, travelers moving across the community could introduce the EVD from one to the other community. There were three imported EVD cases detected in the pilot communities and one of them triggered five secondary cases. Travelers potentially have a longer community infective time after getting Ebola infection because they are commonly harder to monitor [20, 21]. This finding has also been reported in Liberia, where travelers led to reintroduction of EVD in the local EVD-free community [22, 23]. Second, no EVD cases were found among the health care workers in our study, though they have been a highly affected group since the beginning of the EVD outbreak [24–26]. This might reflect the fact that the infection risk among the health care facilities decreased with the improvement of the infection control awareness, and better use of equipment and skills of the health care workers. Furthermore, we found that all nine secondary cases were either household members or neighbors of the Ebola infector, which demonstrated that community members living close to the EVD case have become the most high risk group for Ebola infection. Even in this later stage of the EVD epidemic, we found that some sick persons would conceal their exposure history to EVD cases and thus delay the time for isolation, which showed the complexity of Ebola transmission interruption in the community.

This study had limitations. First, we were not able to follow up specifically on what the trained participants did in their community, and no systematic evaluation of the impact of the widespread community education action on the EVD prevention was conducted. In this emergency situation, we were only able to select very few of the participants of the workshop to perform a simple assessment of their post-training understanding of the EVD prevention messages and how many community persons they shared messages with. Second, we were not able to evaluate the response performance in the three pilot communities by comparing changes in epidemiologic indicators of disease transmission before and after the implementation of intensified measures in the same settings, because we could not get the detailed local epidemiological data before the study period. However, by comparing the three heavily EVD impacted pilot communities with the whole country in Sierra Leone during the same period, we believe our results support our conclusion that community level social mobilization and community engagement were an effective strategy in the special context.

## Conclusion

Our study shows that mobilization of community members engaging in EVD prevention and transmission interruption measures in their community can be an effective way to reach the goal of EVD-free community, even in the context of poor public health infrastructure and human resources. This response strategy against Ebola transmission in the community should be highlighted and the field-operational guidance at the community level should be well developed, tailored and extended nationwide in Sierra Leone, as well as in all the EVD-affected countries in their unique contexts, so as to achieve the EVD-free goal.

## Abbreviations

CDC, Center for Disease Control and Prevention; DSO, Disease Surveillance Officers; EVD, Ebola virus disease; MOHS-SL, Ministry of Health and Sanitation of Sierra Leone; WHO, World Health Organization

## Additional files

Additional file 1: Multilingual abstracts in the six official working languages of the United Nations. (PDF 299 kb) Additional file 2: Technical supplementary for practical community-based response strategy to interrupt Ebola transmission in Sierra Leone, 2014-2015. (DOC 721 kb)
