# Supplementary material for: A practical community-based response strategy to interrupt Ebola transmission in sierra Leone, 2014–2015
Source: Infect Dis Poverty. 2016 Aug 5;5:74. doi: 10.1186/s40249-016-0167-0 (PMC4974705; doi:10.1186/s40249-016-0167-0)
Supplement: Additional file 1: — Multilingual abstracts in the six official working languages of the United Nations. (PDF 299 kb) [file 40249_2016_167_MOESM1_ESM.pdf]

Translation of the abstract into the six official working languages of the United Nations

استراتيجية الاستجابة المجتمعية والعملية للحد من انتقال فيروس ايبولا في سيراليون 2014-2015.

تشونغ جي لي- ون شياو تو-سياو شنغ وانغ- غو كنغ شي- زون دونغ ين- هاي جانسو- تاو شين- دا بنغ تشانغ- جيان دونغ لى- شان ال في- تشون لي تساو- روي-تشيان شيه- هونغ تشو لو- رونغ منغ جيانغ- تشنغ تساو- زاي جي ان- لي لي لي- جي شو- يان ونغ شيونغ- وي زانغ- وي زانغ- هونغ وي تشانغ- وين شين تشن- هوا لينغ- وين شو- جيان كاي- هوان جين لوا- شوا شينغ شينغ- كان جان شينغ- كوانغ وي- شين شو لي- مي لي- هاي جيانغ- لي كوان دينغ- مينغ كوان تشين- شيانغ هوا- فينغ شو- شوا هوي لاي- شي تشن باي- لونغ جي يي- جيان يي ياو- وين ووا ين- جياو جين سان- لين شياو-فو كيانغ ليو- شيانغ كيانغ ليو- هونغ وي فان- زنغ كيانغ كوا- جي كون زو- هاو زانغ- دا شين ني- توماس تي سامبا- كون لي- هونغ جي يو- يو وانغ- شياو فينغ ليانغ.

ملخص

نبذة عن الدراسة: أنتشر مرض فيروس ايبولا بسرعة في غرب افريقيا في عام 2014 مما أدى الى فقدان الآلاف من الأرواح. ويعد اشراك المجتمع المحلي أحد الاستراتيجيات المتبعة لوقف انتقال فيروس ايبولا. وكان هناك حاجة لاستكشاف الاجراءات العملية على مستوى المجتمع في هذا المجال وتصميم هذه الاجراءات لتتلائم مع سياق معين لكل مجتمع.

طرائق البحث: أولا: تم إطلاق حملة التعريف بالمرض على مستوى المجتمع المحلي حول الوقاية من مرض فيروس ايبولا من خلال التعبئة الاجتماعية في ست مناطق في سيراليون ابتداءا من شهر تشرين الثاني من عام 2014. ثم ابتداءا من شهر مايو من عام 2015 في ثلاث مجتمعات أخرى مشاركة في التجربة من خلال تنظيم واشراك اعضاء المجتمع المحلي المدربين بتنفيذ التدابيراللازمة لمنع ووقف مرض فيروس ايبولا من الانتشار من خلال اشراكهم في التقارير عن حالة التأهب والمتابعة والتعبئة الاجتماعية. وتم تقييم المؤشرات الوبائية لوقف انتقال هذا المرض في ثلاث مجتمعات متضمنة في الدراسة.

النتائج: تم تدريب 6016 ناشط اجتماعي في هذه المجتمعات من 185 قسم من خلال عقد 279 ورشة عمل في ست مناطق وكنتيجة تم ايصال الرسالة حول مرض فيروس ايبولا لحوالي 631680 شخص مقيم. وقد تم الابلاغ عن عن 72 حالة لمرض فيروس ايبولا في ثلاث مجتمعات مشاركة في الدراسة. وقد تم الابلاغ عن 70.8% من هذه الحالات من قبل اعضاء مدربين من المجتمع المحلي. وتم تحديد 14 حالة لمرض فيروس ايبولا من الحالات التي تم الابلاغ عنها. وتمكن متابعي حالات المرضى من الكشف عن 64.3% من حالات مرض فيروس ايبولا. وكان متوسط مدة العدوى المجتمعية هي يوم واحد و معدل الهجمة الثانوية كان حوالي 4.2% دون تحفيز جيل ثالث للعدوى. ولم تسجل اي حالة عدوى بين العاملين أو أي عملية دفن غير امنة أو عدم امثال لتدابير ضبط فيروس ايبولا. تم تصميم المعاير المجتمعية هذه للتقليل من عدد حالات الاصابة بفيروس ايبولا بمعدل 77 حالة وقد تم تحقيق التخلص نهائيا من فيروس ايبولا قبل اربعة أشهر في دراسة للمجتمعات وليس سيراليون ككل.

الخاتمة: تعد استراتيجية المجتمعات للتعبئة المجتمعية واشراك المجتمع فعالة في الكشف والتقليل من مدى انتقال عدوى فيروس ايبولا في بلد يعاني من نظام صحي ضعيف. ومن المتحمل ان تكون هذه التجربة العملية الناجحة للتقليل من انتشار فيروس ايبولا في مجتمع يعاني من شح في الموارد فعالة ومفيدة للمجتمع العالمي لمحاربة فيروس ايبولا وغيرها من الأمراض الأخرى في المستقبل.

Translated from English version into Arabic by Randa82, through

TRANSLATORS
WITHOUT BORDERS

2014-2015 年塞拉利昂基于社区防控策略阻断埃博拉病毒病的传播

李中杰，涂文校，王晓春，施国庆，尹遵栋，苏海军，申涛，张大鹏，李建东，吕山，曹淳力，解瑞谦，卢洪洲，蒋荣猛，曹政，安志杰，李雷雷，徐杰，熊衍文，臧炜，张巍，张宏伟，陈文森，凌华，徐闻，蔡剑，罗焕金，邢学森，郑灿军，魏强，李新旭，李美，姜海，邓立权，陈明泉，霍翔，徐峰，来学惠，白熙臣，叶龙杰，姚建义，殷文武，孙校金，肖林，刘富强，刘晓强，范洪伟，寇增强，周吉坤，张昊，倪大新，Thomas T. Samba，李群，余宏杰，王宇，梁晓峰

摘要
引言：2014 年埃博拉病毒病（EVD）在西非快速传播，导致上万人失去生命。社区参与防控是阻断疫情传播的关键策略之一，该策略需要探索切实可行且因地制宜的现场实践措施。
方法：首先，2014 年 11 月起在社区层面开展健康教育，对象包括塞拉利昂 6 个地区的社会动员人员。其次，2015 年 1～5 月在塞拉利昂西区（首都所在区）的 3 个示范区内组织受训的防控人员实施阻断疫情传播的关键防控措施，包括预警病例的发现和报告、流行病学调查、密切接触者追踪和社会动员；同时收集防控效果相关的流行病学指标进行评估。
结果：在塞拉利昂 6 个地区 185 个选区举办了 279 场培训班、共有 6 016 名社会动员人员接受培训，估计有 631680 名当地居民通过这些受训的社会动员人员传播获得 EVD 知识。在研究期间，3 个示范区共报告了 72 名 EVD 预警病例，70.8%是由受训的社区工作人员报告的，其中确诊病例 14 例。64.3%的确诊病例是对密切接触者进一步追踪和健康监测发现的。确诊病例在社区的传染期中位数为 1 天，续发率为 4.2%，没有发生三代传播。社区内无发生医护人员感染，也没有发生不安全丧葬或攻击防控人员事件。通过数学模型估计，比起同期塞拉利昂全国平均的防控水平，在示范区开展的强化防控干预措施，至少可以减少 77 例 EVD 病例，提前 4 个月达到社区内无 EVD 疫情的目标。
结论：基于社区的社会动员和社区参与策略对于卫生体系薄弱的国家发现 EVD 病例并阻断传播是有效的。在资源匮乏的社区实施 EVD 防控的成功经验可能对全球未来防控 EVD 和其他传染病也有借鉴意义。

Translated from English version into Chinese by Wen-Xiao Tu

Stratégie de réponse communautaire pratique pour interrompre la transmission du virus d’Ébola au Sierra Leone, 2014-2015

Zhong-Jie Li, Wen-Xiao Tu, Xiao-Chun Wang, Guo-Qing Shi, Zun-Dong Yin, Hai-Jun Su, Tao Shen, Da-Peng Zhang, Jian-Dong Li, Shan Lv, Chun-Li Cao, Rui-Qian Xie, Hong-Zhou Lu, Rong-Meng Jiang, Zheng Cao, Zhi-Jie An, Lei-Lei Li, Jie Xu, Yan-Wen Xiong, Wei Zang, Wei Zhang, Hong-Wei Zhang, Wen-Sen Chen, Hua Ling, Wen Xu, Jian Cai, Huan-Jin Luo, Xue-Sheng Xing, Can-Jun Zheng, Qiang Wei, Xin-Xu Li, Mei Li, Hai Jiang, Li-Quan Deng, Ming-Quan Chen, Xiang Huo, Feng Xu, Xue-Hui Lai, Xi-Chen Bai, Long-Jie Ye, Jian-Yi Yao, Wen-Wu Yin, Jiao-Jin Sun, Lin Xiao, Fu-Qiang Liu, Xiao-Qiang Liu, Hong-Wei Fan, Zeng-Qiang Kou, Ji-Kun Zhou, Hao Zhang, Da-Xin Ni, Thomas T. Samba, Qun Li, Hong-Jie Yu, Yu Wang, Xiao-Feng Liang

Résumé
Contexte: La fièvre d’Ébola s’est rapidement propagée en Afrique de l’Ouest en 2014 et a causé des milliers de morts. L’engagement des populations a été l’une des stratégies cruciales mises en œuvre pour enrayer la transmission du virus et des mesure communautaires pratiques ont été explorées sur le terrain et adaptées au contexte spécifique de chaque collectivité.
Méthodes: Un travail d’éducation communautaire à la prévention de la fièvre d’Ébola a tout d’abord été lancé dans six districts du Sierra Leone à partir de novembre 2014, afin de former des animateurs sociaux dans ces collectivités. Ensuite, de janvier à mai 2015, des habitants de trois localités pilotes ont été formés et impliqués dans la réalisation de mesures de prévention et d’arrêt de la transmission du virus : alerte et notification de cas, suivi des contacts, mobilisation sociale. Les indicateurs épidémiologiques de l’interruption de la transmission ont été évalués dans les trois collectivités étudiées.
Résultats: Au total, 6016 animateurs sociaux locaux relevant de 185 dispensaires ont été formés au cours de 279 tenus dans les six districts, et on estime que le message concernant le virus est parvenu à 631 680 habitants. Dans

les trois communautés pilotes, 72 alertes de cas de fièvre d'Ébola ont été rapportées, dont 70,8 % par des habitants spécialement formés, et 14 cas de fièvre d'Ébola ont été confirmés. Le suivi des contacts a permis de détecter 64,3 % des cas de fièvre d'Ébola. La durée moyenne de l'infectivité communautaire des cas était d'un jour. Le taux d'attaques secondaires était de 4,2 % et il n'y a pas eu de troisième génération d'infection. Aucun professionnel de la santé n'a été infecté et aucun cas d'inhumation à risques ou de non-respect des mesures de lutte contre le virus d'Ébola n'a été relevé. La modélisation montre que les mesures ont évité 77 cas de fièvre d'Ébola et l'objectif d'élimination de la maladie a été atteint quatre mois plus tôt dans les collectivités étudiées que sur le reste du territoire du Sierra Leone.

Conclusions: La stratégie communautaire de mobilisation sociale et d'implication des habitants s'est avérée efficace pour la détection des cas et pour réduire l'ampleur de la transmission du virus Ébola dans un pays dont le système de santé publique est insuffisant. La réussite de cette expérience pratique de réduction du risque de transmission du virus Ébola dans une communauté disposant de peu de ressources suggère une approche utile pour lutter contre ce virus et contre d'autres maladies dans le monde à l'avenir.

Translated from English version into French by Suzanne Assenat, through

TRANSLATORS
WITHOUT BORDERS

Практическая стратегия реагирования на местном уровне направленных на препятствие передач Эбола в Сьерра-Леоне, 2014-2015

Чжун-Цзе Ли, Вэнь-Сяо Ту, Сяо-Чун Ван Го-Цин Ши, Зун-Донг Инь, Хай-Юнь Су Тао Шен, Да-Пэн Чжан, Цзянь-Донг Ли Шан Лв, Чун-Ли Цао , Руи-Цянь Се, Гонг-Чжоу Лу, Ронг-Мэн Цзян, Чжэн Цао Чжи-Джи Ан, Лей-Лей Ли, Цзе Сюй, Ян Вэнь-Сюн, Вэй Занг, Вэй Чжан, Хун-Вэй Чжан, Уэн- Сен Чен, Хуа Лин, Вэнь Сюй Цзянь Цай, Хуань-Цзинь Ло, Сюэ Шэн Ксинг, Кан-Юнь Чжэн, Цян Вэй, Синь-Сюй Ли, Мэй Ли, Хай Цзян, Ли-Цюань Дэн, Мин-Цюань Чэнь , Сян Хо, Фэн Сюй, Сюэ-Хи Лай, Си-Чэнь Бай, Лонг-Джи Е., Цзянь-Йи Яо, Вэнь-Ву Инь, Цзяо-Цин ВС, Лин Сяо, Фу-Цян Лю Сяо-Цян Лю, Гонг-Вэй Фан, Цзэн-Цян Коу, Джи-Кун Чжоу, Хао Чжан, Да-Кцин Ни, Томас Т. Самба, Цюнь Ли, Гонг-Джи Ю, Ю Ван, Сяо Фэн-Лян

Аннотация

Цель: Болезнь Вирус Эбола быстро распространился в Западной Африке в 2014 году, что привело к потере тысяч жизней. Участие сообщества является одним из ключевых стратегий, направленных на препрятствие передач Эбола, и практические меры на местном уровне должны быть изучены в полевых условиях и с учетом конкретной ситуации общин.

Методы: Во-первых, был запущен образование на местном уровне по прерыванию Геморрагической Лихорадки Эбола (ГЛЭ) для социальных мобилизаторов общины в шести районах в Сьерра-Леоне, начиная с ноября 2014 г. Затем, с января по май 2015 года в трех пилотных общинах, члены общины подтотовленные на местном уровне были вовлечены для участия в реализации мер по профилактике ГЛЭ и прерывании передач, путем вовлечения их в отчет экстренного сообщения, отслеживанию контактов и социальной мобилизации. В трех исследуемых сообществах эпидемиологические показатели прерывания передачи были оценены .

Результаты: В общей сложности 6 016 общинных социальных мобилизаторов из 185 отделений прошли подготовку путем проведения 279 семинаров в шести районах, и ГЛЭ сообщение достигло около 631 680

жителей. В трех пилотных общинах, было зарегистрировано 72 случаев ГЛЭ, 70,8% из них обнаружены обученными членами местных общин, а также 14 случаев ГЛЭ были окончательно определены. Прослеживание контакта   обнаружено 64,3% случаев ГЛЭ. Средняя продолжительность инфекционности населения для случаев составил 1 день. Показатель вторичной поражённости составила 4,2%, а третье поколение инфекции не было вызвано. Ни один медицинский работник не был заражен, и небезопасное погребение и несоблюдение мер сооствтсвия ГЛЭ зафиксировано не было. Меры на уровне общин были смоделированы для снижения 77 случаев ГЛЭ и цель защиты от ГВЭ была достигнута за четыре месяца ранее в учебных сообществах, чем всей стране Сьерра-Леоне.

Выводы: стратегия социальной мобилизации и участия общин территориально было эффективным в выявлении случаев заболевания и снижение степени передачи Эбола на местном уровне со слабой системой здравоохранения. Успешно-практический опыт, в целях уменьшения риска передачи вируса Эбола в сообществе с бедными ресурсами потенциально был бы полезным для международного сообщества в борьбе против ГЛЭ и других заболеваний в будущем.

Translated from English version into Russian by Turdimurot Rakhmonov, through

Una respuesta comunitaria práctica para interrumpir la transmisión del ébola en Sierra Leona, 2014-2015

Zhong-Jie Li, Wen-Xiao Tu, Xiao-Chun Wang, Guo-Qing Shi, Zun-Dong Yin, Hai-Jun Su, Tao Shen, Da-Peng Zhang, Jian-Dong Li, Shan Lv, Chun-Li Cao, Rui-Qian Xie, Hong-Zhou Lu, Rong-Meng Jiang, Zheng Cao, Zhi-Jie An, Lei-Lei Li, Jie Xu, Yan-Wen Xiong, Wei Zang, Wei Zhang, Hong-Wei Zhang, Wen-Sen Chen, Hua Ling, Wen Xu, Jian Cai, Huan-Jin Luo, Xue-Sheng Xing, Can-Jun Zheng, Qiang Wei, Xin-Xu Li, Mei Li, Hai Jiang, Li-Quan Deng, Ming-Quan Chen, Xiang Huo, Feng Xu, Xue-Hui Lai, Xi-Chen Bai, Long-Jie Ye, Jian-Yi Yao, Wen-Wu Yin, Jiao-Jin Sun, Lin Xiao, Fu-Qiang Liu, Xiao-Qiang Liu, Hong-Wei Fan, Zeng-Qiang Kou, Ji-Kun Zhou, Hao Zhang, Da-Xin Ni, Thomas T. Samba, Qun Li, Hong-Jie Yu, Yu Wang, Xiao-Feng Liang

Resumen

Introducción: En el 2014, la enfermedad del virus del ébola se diseminó con rapidez en el oeste de Á frica, y produjo la pérdida de miles de vidas. La participación de la comunidad fue una de las estrategias claves para interrumpir la transmisión de enfermedad. Fue necesario explorar las medidas prácticas comunitarias sobre el terreno, y adaptarlas al contexto específico de cada comunidad.

Métodos: En primer lugar, se lanzó un proyecto de educación comunitaria sobre la prevención de la enfermedad del virus del ébola (EVE) para los movilizadores sociales de la comunidad en seis distritos de Sierra Leona, que comenzó en noviembre de 2014. Luego, entre enero y mayo de 2015, se organizó a miembros locales capacitados en tres comunidades piloto para implementar medidas de prevención e interrupción de la transmisión de EVE. Para esto, se los involucró en informes de casos de alerta, la trazabilidad de contactos y la movilización social. Se evaluaron los indicadores epidemiológicos de interrupción de la transmisión en tres comunidades de estudio.

Resultados: Un total de 6 016 impulsores sociales comunitarios de 185 servicios fueron entrenados por medio de la realización de 279 talleres en los seis distritos. Se estima que el mensaje sobre EVE alcanzó a 631 680 residentes. En tres comunidades de prueba se reportaron 72 casos de alerta de EVE de los cuales, el 70,8% fueron detectados por miembros capacitados de la comunidad local. Al final, se identificaron 14 casos de EVE. La

trazabilidad de contactos detectó el 64,3% de los casos de EVE. La duración promedio por caso de infecciosidad en la comunidad fue de un día. La tasa de ataque secundario fue 4,2% y no se desencadenó ninguna infección de tercera generación. Ningún trabajador sanitario resultó infectado, tampoco se registró que se llevaran a cabo entierros en condiciones inseguras, ni incumplimientos de las medidas de control de EVE. Las medidas comunitarias fueron diseñadas para reducir 77 casos de EVE, y el objetivo de eliminación de EVE se logró cuatro meses antes en las comunidades de estudio que en el resto de Sierra Leona.

Conclusiones: La estrategia comunitaria de movilización social y participación comunitaria resultó ser eficaz en lo que respecta a la detección de casos, y a la reducción del alcance de la transmisión del ébola en un país con un sistema de salud precario. La exitosa experiencia práctica de reducir el riesgo de transmisión de ébola en una comunidad con escasos recursos podría ser útil para la comunidad mundial en la lucha contra la EVE y otras enfermedades en el futuro.

Translated from English version into Spanish by Mpgorgone, through

TRANSLATORS
WITHOUT BORDERS
