# Supplementary material for: A practical community-based response strategy to interrupt Ebola transmission in sierra Leone, 2014–2015
Source: Infect Dis Poverty. 2016 Aug 5;5:74. doi: 10.1186/s40249-016-0167-0 (PMC4974705; doi:10.1186/s40249-016-0167-0)
Supplement: Additional file 2: — Technical supplementary for practical community-based response strategy to interrupt Ebola transmission in Sierra Leone, 2014-2015. (DOC 721 kb) [file 40249_2016_167_MOESM2_ESM.doc]

**Supplementary Appendix**

**Table of Contents**

| **Category** | **Contents** | **Page** |
| --- | --- | --- |
| **File S1** | **The framework of community-based response strategy against Ebola in Sierra Leone, 2014-2015.** | **1** |
| **File S2** | **The operational proposal of intensified surveillance and response in the three communities in Sierra Leone** | **2** |
| **File S3** | **Methods of estimating the Number of Ebola Cases among the three communities of Western Area Rural, Sierra Leone** | **20** |
| **File S4** | **Description of Ebola cases cluster in the three pilot communities** | **24** |

**Supplementary Appendix File S1**

**The framework of practical community-based response strategy to interrupt Ebola transmission** in Sierra Leone, 2014-2015.

| **Objective** | **Scope** | **Duration** | **Measures** |
| --- | --- | --- | --- |
| **Part one: Massive community-level education in six districts** | | | |
| To enhance the awareness of Ebola prevention, and promote social mobilization, and social engagement at community level. | Six districts ( totally 14 districts in Sierra Leone), affected heavily by Ebola. | From 11^st^, November, 2014 to 5^th^, Febuary 2015 | - To advocate the community leadership and social mobilization. - To provide the face-to-face training courses for the community leaders, community activist, social mobilizor on the Ebola health message. - To widespread distribute the health promotion materials into each community by the trained community members. - To encourage the trained community persons to spread the Ebola key message to the persons in their community, i.e. family members, relatives, neighbors, friends, and the others. |
| **Part two: Intensified surveillance and response in three pilot communities** | | | |
| To interrupt the Ebola transmission in the community with enhanced case detection and rapid response actions by involving in the local community members. | Three communities (Jui, Kossoh Town, and Grafton) located in the Western Area Rural District, with high risk of Ebola introduction and local transmission. | From 13^rd^ January, 2015 to 19^th^, May, 2015 | - To build community-based Ebola response team against Ebola in the community. - To enhance active case detection action. - To facilitate rapid response to the Ebola alert case in the community. - To shorten the community infectively time of Ebola case. - To strictly implement the contact identification and contact tracing . - To ensure the safe burial in the community. - To build up the incentive and practical operational mechanism for the community’s Ebola health response team. |

**Supplementary Appendix File S2**

**Operational Proposal** **on Intensified Surveillance and Response to Ebola**

**in Three Communities of Western Area Rural District, Sierra Leone**

**1. Background**

From November 2014 to May 2015, the Chinese Center for Disease Control and Prevention (China CDC) had dispatched four public health teams, with 53 staffs, to assist in Ebola outbreak control in Sierra Leone. With aim to stop the EVD spreading in the community, Chinese public health experts in Sierra Leone, jointly with Ministry of Health and Sanitation and District Health Management Team of Sierra Leone, has launched an integrated community-based response strategy for Zero-Ebola in three pilot communities with high risk of Ebola transmission since January, 2015. The Jui, Kossoh town, and Grafton communities in the Western Area Rural District, with high risk of Ebola introduction and transmission in the community, were selected as the field sites of this intensified program, which are located in the south-eastern part of the capital city, Freetown, with a total of more than 9 thousand of households and 42.7 thousand of residents.

**2. Purposes**

To strengthen the implementation measures of case detection, investigation, isolation, social mobilization and community engagement for Ebola in the three communities, and reach the goal of Zero-Ebola in the community as early as possible.

**3. Case definitions**

The case definition related to Ebola surveillance and response is in accordance with the guideline issued by World Health Organization and Ministry of Health and Sanitation of Sierra Leone [1, 2].

**3.1. Alert case**

Illness with onset of fever and no response to treatment of usual causes of fever in the area, OR at least one of the following signs: bleeding, bloody diarrhea, bleeding into urine, OR any sudden death.

**3.2 Suspected case**

Any person, alive or dead, suffering or having suffered from a sudden onset of high fever and having had contact with a suspected, probable or confirmed Ebola case.

**OR**

any person with sudden onset of high fever and at least three of the following symptoms:

• headaches

• vomiting

• anorexia / loss of appetite

• diarrhea

• lethargy

• stomach pain

• aching muscles or joints

• difficulty swallowing

• breathing difficulties

• hiccups

**OR**

any person with inexplicable bleeding

**OR**

any sudden inexplicable death

**3.3 Probable case**

Any suspected case evaluated by a clinician.

**OR**

Any deceased suspected case (where it has not been possible to collect specimens for laboratory confirmation) having an epidemiological link with a confirmed case.

**3.4 Confirmed case**

Any suspected or probably cases with a positive laboratory result. Laboratory confirmed cases must test positive for the virus antigen, either by detection of virus RNA by reverse transcriptase-polymerase chain reaction (RT- PCR), or by detection of IgM antibodies directed against Ebola.

**3.5 Non-case**

Any suspected or probable case with a negative laboratory result for Ebola from specimen collected ≥72 hours after symptom onset.

**3.6 Case contact**

Any person having been exposed to a suspect, probable or confirmed case of Ebola in at least one of the following ways:

- has slept in the same household with a case

- has had direct physical contact with the case (alive or dead) during the illness

- has had direct physical contact with the (dead) case at the funeral

- has touched his/her blood or body fluids during the illness

- has touched his/her clothes or linens

- has been breastfed by the patient (baby)

Provided that this exposure has taken place less than 21 days before the identification as a contact by surveillance teams.

**4. Enhanced Surveillance and Response Measure**

**4.1 Active detection of alert cases**

Four major active case detection measures were implemented in the three communities:

**(1) Contact tracing:** to find the alert Ebola cases among the contact persons being registered and under following up.

**(2) House-to-house visits:** to assign a small health team with 2-3 persons to perform daily house-to-house visit for the community households to identify persons who are feeling unwell for any reason and any death from any cause, as well as to search for the situation meeting with the definition of Ebola trigger event.

**(3) Health facility reports:** to record the report from the community’s health facility (peripheral health unit, PHU), when the visiting patients meeting with the definition of Ebola alert case.

**(4) Community reports:** to detect the alert case via community report by community leader, family member, neighbor, or others.

Any person, who meet with the definition of Ebola alert case, should be report to the Ebola command center via calling 117 hot line or report directly to the relevant disease surveillance officers (DSO) at the community level.

**4.2 Alert case verification**

After receiving the report of alert case, the Ebola Alert center should fill in the Call Center Reporting Form (form 1) to record the information on the alert case, and distribute it to the local relevant DSO as soon as possible. The DSO should finish on-site verification on the alert case within 24 hours of getting notification, to make preliminary judgment on whether the case is a suspected Ebola case or not. The outcome of verification for each alert case and the daily summary would be recorded in the form 2 and form 3.

**4.3 Suspected case investigation, sampling and isolation**

Once the alert case was identified as the suspected case, the case investigation form (form 4) should be filled by DSO via field interviewing with the case. Meanwhile, the specimen of the suspected case would be sampled and sent to the corresponding Ebola test laboratory. The suspected case should be removed outside of the community to the Ebola Holding Center (EHC) for proper isolation until getting the laboratory test result. And all the contact persons exposed to this suspected cases would be listed, and their information were recorded (form 5).

**4.4 Contact tracing**

Once the suspected case was identified as the probable or confirmed Ebola case, the case should be transferred to be treated and managed in the Ebola Treatment Unit (ETU), and the DSO shall immediately send the Viral Hemorrhagic Fever Contact Listing Form (form 5) to the contact tracer team. After receiving the contact list, the contact tracer on-duty will trace all contacts on the name list. All the contacts shall be quarantined at home or in a designated place for consecutive 21 days after their last exposure to the probable or confirmed patient. The contact tracer shall daily visit the contacts twice a day, and fill in the contact tracing form (form 6). The contact tracer supervisor should record the contact daily monitoring form (form 7), and report to the Surveillance Alert center or the local DSO once the alert symptoms are found among the contact persons [3].

**4.5 Safe burial**

In the Western Area of Sierra Leone, the professional burial teams were led directly by the Ministry of Health and Sanitation, which were in charge of conducting the dignified and safe medical burial. In the three pilot communities, all deaths with any kind of reason, including community deaths and deaths occurring at health facilities, should be reported to the burial team [4]. Subsequently, all deaths will be classified as a confirmed, probable, suspect, or not a case. In no or low transmission areas, only bodies determined to be not a case by the investigator may be left with the family for community burial. Bodies that are identified as suspect, probable, or confirmed will be collected and buried by the professional burial team. Coordinators and social mobilizers of the affected community shall, before, during and after the funeral, assist the safe burial with health education and psychological comfort for relatives, neighbors and other community members of the deceased and reduce community exposure.

**4.6 Social mobilization and community engagement**

Thirty social mobilization teams, consisting of the local community or religious leaders and members, were established to daily visit and screen all residential households to detect the abnormal events and patients in the three pilot communities, as well as to educate the local residents on Ebola related knowledge and promote health behaviors by distributing the leaflets to each household and putting on the posters in the community. Social mobilizers shall support suspected case households on temporary home quarantine, publicize the knowledge on how to prevent Ebola among the suspected patient’s relatives and neighbors, and give psychosocial and mental health support. At the same time, billboards and banners on Ebola prevention and case reporting were set up by the roadsides through three communities. The main topics of social mobilization included Ebola alert case report, Ebola transmission route, home sanitation, safe burial practices, and reduction of stigma, which are intended to create and enhance public awareness about Ebola, the risk factors for its transmission, its prevention and control among the people.

**5. Working mechanism to support the enhanced surveillance and response measure**

**5.1 Effective coordination and close collaboration with stakeholders**

The stakeholders related to the Ebola response in the pilot communities were involved. The intensified surveillance and response strategy in the three pilot communities was approved by Ministry of Health and Sanitation of Sierra Leone, and memorandum of understanding was signed between Chinese Center for Disease Control and Prevention and District Health Management Team (DHMT) of Western District. The implementation proposal and work mechanism were jointly developed by China public health team and DHMT of Western District, which is in accordance with the guidance of WHO. The key persons from DHMT, including District Medical Officer, surveillance officer, and head of social mobilization, and the community leaders, played the role of field coordination, staffs recruitment, supervision, and technical assistance. The working mechanism was effectively established among the Peripheral Health Units in the three communities and the Ebola Holding Center (EHC), Ebola Treatment Unit (ETU) and the Labs for Ebola test.

**5.2 Scaling-up the human resources for Ebola response in the community**

As the reason of poor human resources for Ebola response at the community level, it’s essential to scale up the response taskforce with required quality engaging in the Ebola control in the community. The local community’s and religious leaders and activists, who have a high school and higher education level and can be taught to follow the response proposals, were recruited and then trained on the skill of social mobilization, case report and investigation, and contact tracing, which including disease surveillance officer, contact tracer, social mobiliser, and field supervisor, etc. Totally 101 staffs were employed, including 2 senior coordinators, 3 community coordinators, 5 senior supervisors, 3 support staffs, 5 field supervisors, 5 disease surveillance officers, 18 contact tracers, and 60 social mobilizers.

**5.3 Full coverage of response taskforces in the community**

A “sector” approach, which divided the three pilot communities into 30 subsections, was taken to ensure all the community households and residents were fully covered, by means of assigning 15 social mobilization teams, 15 contact tracing teams, 5 DSO teams, and 3 field supervising teams to the corresponding designated subsections (Table S1).

**5.4 Ensuring effective implementation of the control measures in the community**

The responsibility of each work team was clearly determined, by quantifying the requirement of each response action, i.e. at least 40 households should be visited daily by each social mobilize team, 100% of the alert cases should be investigated with 24 hours after getting report by the disease surveillance officer, all the contacts should be registered and followed tracing daily for the whole 21 days by the community contact tracer, etc. Weekly work schedule for case surveillance and contact tracing team was established (form 8), which required the field teams in the community to report their work and submit the filled investigation forms per day, and their work would be reviewed timely. The field implementation of control measures would be evaluated and discussed on the routine weekly meeting, involving in the participants from the field work teams, staffs of DHMT and China public health team.

**5.5 Building the incentive mechanism for the recruited health staffs**

Incentive mechanism was built up for the community response taskforce, by providing the allowance for their time contribute to the Ebola responding work. The extra bonus would be provided to the staffs in the weekly meeting who have done their job well, i.e. stop the unsafe burial in the community, timely report the unexplainable death or the cluster of illness in the household, etc.

**
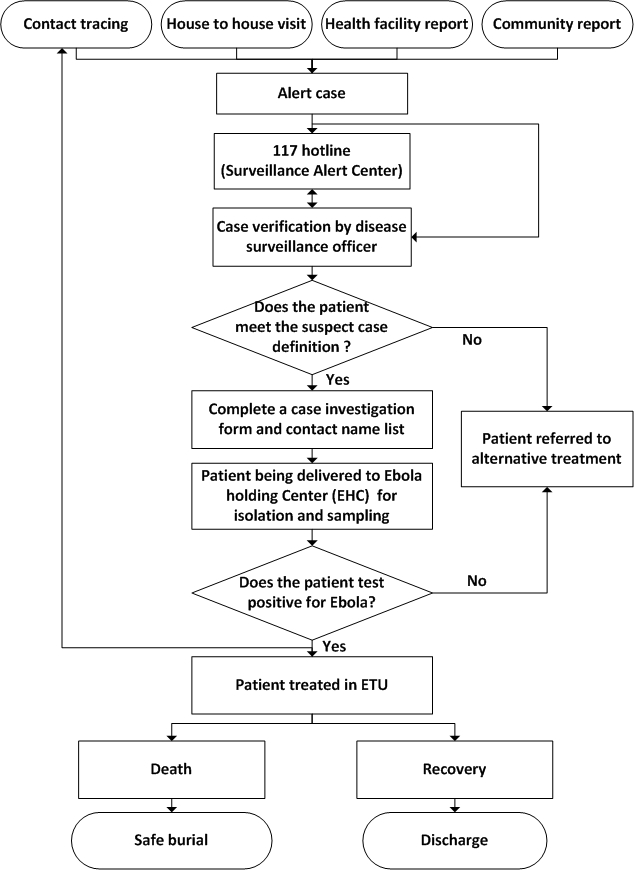
**

**Figure S2_1. Operational workflow of Ebola case detection, investigation and management in the three pilot communities, Sierra Leone.**

**Table S2_1. The assignment of community response taskforces for Ebola in the three communities with full-coverage of subsection, Sierra Leone.***

| **Community name** | **PHU name** | **Subsection name** | **Social mobilization team** | **Contact tracing team** | **Disease surveillance officer (DSO) team** | **Field supervisor (FS) team** |
| --- | --- | --- | --- | --- | --- | --- |
| Jui community | Jui Police clinic | Kashew farm | SM team 1 | CT team 1 | DSO team 1 | FS team 1 |
|  |  | Kobba farm | SM team 2 |  |  |  |
|  |  | Jui Police barracks | SM team 3 | CT team 2 |  |  |
|  |  | White house/poultry |  |  |  |  |
|  |  | Lordep | SM team 4 | CT team 3 |  |  |
|  | Jui TECT | Bible Colledge | SM team 5 | CT team 4 | DSO team 2 |  |
|  |  | Barried/Susu Quarter |  |  |  |  |
|  |  | O/C Quarter | SM team 6 |  |  |  |
|  |  | New Site |  | CT team 5 |  |  |
|  |  | Talaban | SM team 7 |  |  |  |
|  |  | Huntingdon | SM team 8 |  |  |  |
|  |  | Jui Hanga | SM team 9 | CT team 6 |  |  |
|  |  | Kambia | SM team 10 |  |  |  |
| Kossoh Town | New London MCHP | Kossoh Town | SM team 11 | CT team 7 | DSO team 3 | FS team 2 |
|  |  | Moko Town | SM team 12 |  |  |  |
|  |  | Youth Farm | SM team 13 | CT team 8 |  |  |
|  |  | New London | SM team 14 |  |  |  |
|  |  | Botton Mango | SM team 15 | CT team 9 |  |  |
| Grafton Community | Grafton Community Health Center | Grafton Police Barracks | SM team 16 | CT team 10 | DSO team 4 | FS team 3 |
|  |  | Sorie Bubu/ Ten House | SM team 17 |  |  |  |
|  |  | Scout Camp | SM team 18 | CT team 11 |  |  |
|  |  | Chesul |  |  |  |  |
|  |  | Gibo Town |  |  |  |  |
|  |  | Looking Town | SM team 19 | CT team 12 |  |  |
|  |  | Grafton Town/Soprus | SM team 20  SM team 21 |  |  |  |
|  |  | New Camp | SM team 22  SM team 23  SM team 24  SM team 25  SM team 26 | CT team 13 |  |  |
|  |  | Wureh Town | SM team 27 |  |  |  |
|  | Heart And Hand | 4 Root/PVA | SM team 28 | CT team 14 | DSO team 5 |  |
|  |  | Part of Old Camp/War Wounded | SM team 29 |  |  |  |
|  |  | Gibike Junction/New Site | SM team 30 | CT team 15 |  |  |

*: Social mobilisers included 30 community teams with 2 staffs for each team; Disease surveillance officers included 5 teams with 1-2 staffs for each team; Contact tracers included 15 teams with 1-2 staffs for each team; Field supervisor included 3 teams and 2 persons for each team. Abbreviation: PHU-peripheral health unit, SM-social mobiliser, CT-contact tracer, DSO-disease surveillance officer, FS-field supervisor.

**Form 1**

**Western area emergency operations center**

Call center reporting form

| **Name of call center operator** |  |
| --- | --- |
| Date |  |
| Time of call |  |
| **INFORMANT DETAIL** |  |
| Name of informant |  |
| Contact number |  |
| Contact address |  |
| **CONTACT DETAIL** |  |
| Name of Suspected person |  |
| Contact address |  |
| Contact phone number |  |
|  |  |
| **History** |  |
| Date of onset of symptoms |  |
| Type of symptoms |  |
| Any other relevant information |  |
|  |  |
| **Surveillance response details** |  |

**Form 2**

**DSO INVESTIGATION LOG**

**(to be used during Ebola Suspect Case Investigation – Western Area, Sierra Leone)**

DSO Name:_____________________________ Today’s Date (DD/MM/YY)_____________________

| **#** | **First Name, Last Name** | **Address** | **False (F), False Repeat (FR), Pending (P), or Unable to Investigate (U)** | **Reason for Alert Code** |
| --- | --- | --- | --- | --- |
| 1 |  |  |  |  |
| 2 |  |  |  |  |
| 3 |  |  |  |  |
| 4 |  |  |  |  |
| 5 |  |  |  |  |
| 6 |  |  |  |  |
| 7 |  |  |  |  |
| 8 |  |  |  |  |

Alert Definitions

**Repeat-**A Person who meets the case definition for Ebola AND has been reported (i.e. CIF completed).

**False** – A Person who has NO Ebola symptoms OR a person who has died (corpse). Note: A death (corpse) is to be coded as a “false alert” for live case surveillance only but will be followed up by the Burials team and could be an Ebola case.

**False Repeat** - A person who has already been investigated and identified as a false alert. (The coding of FALSE remains unchanged)

**Pending** – A person with Fever and LESS THAN THREE Ebola symptoms OR person(s) who DSO was unable to investigate (Note: while NOT enough to meet the case definition, this could be an Ebola case in early phase and symptoms may change over the next days).

**PENDING ALERTS MUST BE INVESTIGATED BEFORE YOU CAN REMOVE THEM FROM YOUR INVESTIGATION LIST.**

**REPEAT ALERTS (INCLUDING FALSE REPEATS) MUST BE INVESTIGATED UNLESS A CASE INVESTIGATION FORM WAS PREVIOUSLY COMPLETED, or a DEATH RECORDED.**

**Form 3**

**Daily Surveillance Summary Form for Ebola Outbreak**

**Area: Date: Team leader:**

**Team Members: 1. 2. 3.**

| **No. of Alerts Received from Alert Team at Start of Today** | **No. of Alerts Added While in the Field** | **Total Alerts Today** | **No. of Alerts investigated** | **No. of Alerts that Met Case Definition**  (Fill out Case Report information Below) | **No. of Repeated Alerts** | **No. of Alerts False/False Repeat Alerts*** | **No. of Pending Alerts*** | **No. of Alerts Unable to investigate Today*** |
| --- | --- | --- | --- | --- | --- | --- | --- | --- |
|  |  |  |  |  |  |  |  |  |

*****False/False Repeat Alerts, Pending Alerts and “Unable to investigate” Alerts MUST be listed on the DSO investigation Log

Submit all DSO investigation Logs for the team when submitting your Daily Summary on Saturday

| **Case NO.** | **Case Name** | **Case Address** | **Sent to isolation/Holding Centre?(Yes/No)** | **Completed Case Report Form?(Yes/No)** | **Line Listed?(Yes/No)** | **Comments** |
| --- | --- | --- | --- | --- | --- | --- |
|  |  |  |  |  |  |  |
|  |  |  |  |  |  |  |
|  |  |  |  |  |  |  |
|  |  |  |  |  |  |  |
|  |  |  |  |  |  |  |

**Any Questions or Concerns from the Team:**

**Form 4**

**Form 5**

VIRAL HEMORRHAGIC FEVER CONTACT LISTING FORM

| **Case Information** | | | | | | | | | |
| --- | --- | --- | --- | --- | --- | --- | --- | --- | --- |
| **Case ID** | **Surname** | **Other Names** | **Head of Household** | **Village** | **Sub-Country** | **District** | **Date of Symptom Onset** | **Date of Admission to Isolation** | **Date of Death** |
|  |  |  |  |  |  |  |  |  |  |

**For all information on location ,please list information on where the contact will be residing for the next month.

| **Contact Information** | | | | | | | | | | | | | |
| --- | --- | --- | --- | --- | --- | --- | --- | --- | --- | --- | --- | --- | --- |
| **Surname** | **Other name** | **Sex**  **(M/F)** | **Age**  **(yrs)** | **Relation to Case** | **Date of Last Contact with Case** | **Type of Contact**  **(1/2/3/4)***  **List all** | **Head of Household** | **Village** | **District** | **Sub-County** | **Village Lead** | **Phone Number** | **Healthcare Worker(Y/N) if yes, what facility?** |
|  |  |  |  |  |  |  |  |  |  |  |  |  |  |
|  |  |  |  |  |  |  |  |  |  |  |  |  |  |
|  |  |  |  |  |  |  |  |  |  |  |  |  |  |
|  |  |  |  |  |  |  |  |  |  |  |  |  |  |
|  |  |  |  |  |  |  |  |  |  |  |  |  |  |
|  |  |  |  |  |  |  |  |  |  |  |  |  |  |
|  |  |  |  |  |  |  |  |  |  |  |  |  |  |

*** Types of Contacts:**

1 = Touched body fluids of the case (blood, vomit, saliva, urine, feces)

2 = Had direct physical contact with the body of the case (alive or dead)

3 = Touched or shared the linens, clothes, or dishes /eating utensils of the case

4 = Slept, ate, or spent time in the same household or room as the case

Contact sheet filled by: Name: Position: Phone:

**Form 6**

2015

**Form 7**

| **DHMT EVD Contact Daily Monitoring** | | | | | | | | | | | | | | | | | | | |
| --- | --- | --- | --- | --- | --- | --- | --- | --- | --- | --- | --- | --- | --- | --- | --- | --- | --- | --- | --- |
| **SN** | **Date of report** | **Name of Contact Tracer Supervisor** | **Urban/Rural** | **Wards** | **Name of PHU** | **Number of houses visited** | | **Number of contacts** | | | **# of contacts seen yesterday** | **# of contacts not seen yesterday** | **# of contacts lost to follow up** | **# of contacts developed symptoms** | **# of contacts finished 21 days** | **Number of Deaths** | | **% of contacts seen** | **comments** |
|  |  |  |  |  |  | **Quarantined houses** | **Non quarantined house** | **Existing** | **New** | **Total** |  |  |  |  |  | **More than one days** | **New** |  |  |
|  |  |  |  |  |  |  |  |  |  |  |  |  |  |  |  |  |  |  |  |
|  |  |  |  |  |  |  |  |  |  |  |  |  |  |  |  |  |  |  |  |
|  |  |  |  |  |  |  |  |  |  |  |  |  |  |  |  |  |  |  |  |
|  |  |  |  |  |  |  |  |  |  |  |  |  |  |  |  |  |  |  |  |
|  |  |  |  |  |  |  |  |  |  |  |  |  |  |  |  |  |  |  |  |
|  |  |  |  |  |  |  |  |  |  |  |  |  |  |  |  |  |  |  |  |
|  |  |  |  |  |  |  |  |  |  |  |  |  |  |  |  |  |  |  |  |
|  |  |  |  |  |  |  |  |  |  |  |  |  |  |  |  |  |  |  |  |
|  |  |  |  |  |  |  |  |  |  |  |  |  |  |  |  |  |  |  |  |
|  |  |  |  |  |  |  |  |  |  |  |  |  |  |  |  |  |  |  |  |
|  |  |  |  |  |  |  |  |  |  |  |  |  |  |  |  |  |  |  |  |
|  |  |  |  |  |  |  |  |  |  |  |  |  |  |  |  |  |  |  |  |
|  |  |  |  |  |  |  |  |  |  |  |  |  |  |  |  |  |  |  |  |
|  |  |  |  |  |  |  |  |  |  |  |  |  |  |  |  |  |  |  |  |
|  |  |  |  |  |  |  |  |  |  |  |  |  |  |  |  |  |  |  |  |
|  |  |  |  |  |  |  |  |  |  |  |  |  |  |  |  |  |  |  |  |
|  |  |  |  |  |  |  |  |  |  |  |  |  |  |  |  |  |  |  |  |
|  |  |  |  |  |  |  |  |  |  |  |  |  |  |  |  |  |  |  |  |

**Form 8**

**Weekly work schedu**le for case surveillance and contact tracing

| **Working Group** | **Monday** | **Tuesday** | **Wednesday** | **Thursday** | **Friday** |
| --- | --- | --- | --- | --- | --- |
|  | **9:00-10:00** | **9:00-10:00** | **9:00-10:00** | **9:00-10:00** | **9:00-10:00** |
| **DSO** | To submit the completed Form 1-5 of three days (Friday, Saturday and Sunday) to program office | To submit the completed Form 1-5 of the last day to program office | | | |
|  | **/** | | | | To attend the weekly meeting at program office |
| **Supervisor of contact tracing** | To submit Form 6 after checking to program office | **/** | | | |
|  | To submit the completed Form 7 of three days (Friday, Saturday and Sunday) to program office | To submit the completed Form 7 of the last day to program office | | | |
|  | To take the blank Form 6-7 from program office | | | | |
|  | **/** | | | | To attend the weekly meeting at program office |
| **Contact Tracer** | 1.To fill in the form_6  2.To Submit the completed (21days) Form 6 to Supervisor for checking each Friday | | | | |
|  | **/** | | | | To attend the weekly meeting at program office |

**Form 1.** Western Area Emergency Operations Center Call Center Reporting Form; **Form 2.** DSO Investigation Logto be used during Ebola Suspect Case Investigation – Western Area, Sierra Leone); **Form 3.** Daily Surveillance Summary Form for Ebola Outbreak; **Form 4.** EBOLA CASE INVESTIGATION FORM - Sierra Leone; **Form 5.** VIRAL HEMORRHAGIC FEVER CONTACT LISTING FORM; **Form 6.**Ministry of Health and Sanitaion Sierra Leone:Contact Tracing Form for Ebola Outbreak; **Form 7.** DHMT EVD Contact Daily Monitoring.

**References**

1. World Health Organization. Case definition recommendations for Ebola or Marburg Virus Diseases, as of 09 August 2014. 2015 (http://www.who.int/csr/resources/publications/ebola/ebola-case-definition-contact-en.pdf?ua=1).

2. WHO Regional Office for Africa. Contact Tracing During an Outbreak of Ebola Virus Disease. Brazzaville. 2014. 2015 (http://www.afro.who.int/en/clusters-a-programmes/dpc/epidemic-a-pandemic-alert-and-response/epr-highlights/4318).

3. Ministry Of Health And Sanitation SL. Sierra Leone Emergency Management Program Standard Operating Procedure for Contact Tracing. 2014.

4. The Emergency Operations Centre of Sierra Leone. Sierra Leone Emergency Management Program Standard Operating Procedure for Safe, Dignified Medical Burials. Version 1, 2014.

**Supplementary Appendix File S3**

**Method of estimating the transmission of Ebola cases in three pilot communities with no intensified response strategy, Sierra Leone**

1. **Objective**

To estimate the duration and size of Ebola transmission in three pilot communities, under the assumption of no intensified response strategy being implemented during the study period of January13, 2015 to July 17, 2015.

1. **Simulation model**

We adopted a spreadsheet-based simulation model developed by United States Center for Disease Control and Prevention (US CDC) in 2014, which is a susceptible, incubation, infectious, recovery (SIIR) Model.^1^ The model allows to estimate the number of Ebola Virus Disease (EVD) cases in a community, tracks patients susceptibility to disease through infectivity, incubation, recovery, and death, and calculates the spread of EVD and its impact for 300 days. The timeline of 300 days is longer than most of the previously recorded outbreaks of EVD.

The model tracks patients through the following states of Ebola-related infection and disease: susceptible to disease, infected, incubating, infectious, and recovered. The infectious state also includes persons who die but whose burial provides risk for onward transmission. The risk associated with unsafe burial is part of the total daily risk for transmission for the patients at home without effective isolation. All infected persons were assumed to eventually become symptomatic.

Patients were categorized into three levels: 1) hospitalized in an Ebola treatment unit (ETU) or medical care facility, 2) home or in a community setting such that there is a reduced risk for disease transmission (including safe burial when needed), and 3) home with no effective isolation.

1. **Source of parameters**

Most of the parameters of EVD transmission in our estimating were the same as US CDC^1^, except the infectious period was updated according to the latest review.^2^ The population of three communities, initially infected cases, and imported cases were from the field work by the intensified response program.

We assumed there were enough ETC beds in the three communities during our study period. Once case from registered contacts was detected, he/she would be isolated in ETC for treatment. However, if the case was not detected from registered contacts, he/she would be isolated very late after onset (7-13 days later), or dying, or even dead at home. And we also found there were no effective isolation at home or in a community setting. So we took the percent of hospitalized cases as the percent of new confirmed cases from registered contacts, and the other cases were with no effective home isolation. These data were obtained or calculated from the indicator of percent of new confirmed cases from registered contacts in the weekly situation report issued by WHO.^3^

The detailed data source of each parameter were listed in the below table.

Table F3_1. The parameters for estimating the number of Ebola cases among three communities of Western Area Rural, Sierra Leone.

| **Parameter** | | **Value** | | | **Data source** |
| --- | --- | --- | --- | --- | --- |
| Human Population | | 42749 | | | Field survey data in the three during the study period. |
| Number of Initially Infected | | 2 | | | Since 13 Jan 2015, there were 2 cases originated from communities: case 1, from Jui, male ,30 years, traditional herbalist, onset date 27 January 2015.  Case 2, female, 23 years, nurth student under training, onset date 1 February 2015. |
| Days People are Infectious | | 7 | | | The latest review in 2015.^2^ |
| Incubation (Days) | | 6.3±3.31 | | | The same as paper published by US CDC |
| Transmission Risk: Daily | Hospitalized | 0.02 | | |  |
|  | Effective home isolation | 0.03 | | |  |
|  | No Effective home isolation | 0.3 | | |  |
| Imported cases | 13 Jan-12 Feb | 1 | | | Case from Grafton, female, 22 years, petty trader, onset date 2 February 2015 |
|  | 13 Feb-15 Mar | 2 | | | Case 1, 23 years, female, from Kossoh Town, onset 18 February 2015, died at home on 25 February 2015. |
|  |  |  |  |  | Case 2, 30 years, female, lived in the urban, went to Jui on 2 March 2015 after onset. |
| Proportion of 3 group cases | | average | min | max |  |
| 13 Jan-12 Feb | Hospitalized | 0·35 | 0·21 | 0·54 | WHO weekly situation report. |
|  | Effective home isolation | 0·00 | 0·00 | 0·00 |  |
|  | No Effective home isolation | 0·65 | 0·79 | 0·46 |  |
| 13 Feb-15 Mar | Hospitalized | 0·49 | 0·18 | 0·78 |  |
|  | Effective home isolation | 0·00 | 0·00 | 0·00 |  |
|  | No Effective home isolation | 0·51 | 0·82 | 0·22 |  |
| 16 Mar-15 Apr | Hospitalized | 0·74 | 0·56 | 0·84 |  |
|  | Effective home isolation | 0·00 | 0·00 | 0·00 |  |
|  | No Effective home isolation | 0·26 | 0·44 | 0·16 |  |
| 16 Apr - 16 May | Hospitalized | 0·45 | 0·22 | 1·00 |  |
|  | Effective home isolation | 0·00 | 0·00 | 0·00 |  |
|  | No Effective home isolation | 0·55 | 0·78 | 0·00 |  |
| 17 May - 16 Jun | Hospitalized | 0·65 | 0·50 | 0·80 |  |
|  | Effective home isolation | 0·00 | 0·00 | 0·00 |  |
|  | No Effective home isolation | 0·35 | 0·50 | 0·20 |  |
| 17 Jun - 17 Jul | Hospitalized | 0·62 | 0·50 | 0·79 |  |
|  | Effective home isolation | 0·00 | 0·00 | 0·00 |  |
|  | No Effective home isolation | 0·38 | 0·50 | 0·21 |  |

**References**

1. Meltzer M I, Atkins C Y, Santibanez S, et al. Estimating the future number of cases in the Ebola epidemic--Liberia and Sierra Leone, 2014-2015. MMWR Surveill Summ 2014;63 Suppl 3:1-14.

2. Velasquez GE, Aibana O, Ling EJ, Diakite I, Mooring EQ, Murray MB. Time from infection to disease and infectiousness for Ebola virus disease, a systematic review. Clin Infect Dis 2015;61(7):1135-40.

3. World Health Organization. Situation reports with epidemiological data: archive. 2015 (http://apps.who.int/ebola/en/current-situation/ebola-situation-report).

**Supplementary Appendix File S4**

**Description of the 14 Ebola cases in three pilot communities, Sierra Leone, 13^rd^, January to 19^th^, May, 2015**

| **Case No.** | **Age& sex** | **Occupation** | **Community** | **Detection source** | **Sporadic case or cluster** | **Death**  **(Y/N)** | **Safe Burial**  **(Y/N)** | **Contact persons** | **His/her secondary cases** | **Epidemiological relationship and Exposure history** |
| --- | --- | --- | --- | --- | --- | --- | --- | --- | --- | --- |
| C1 | 40  M | Petty trader | Jui | Contact persons under quarantine | Cluster 1 | Y | Y | 0 | 1 | C1, the husband of the former identified Ebola confirmed case (C0), was last exposed to C0 on 11^st^ January and was monitored as contact person. C1 was sick and identified as confirmed Ebola cases on 23rd January, and finally died on 24^th^, January, 2015. |
| C2 | 30  M | Herbalist | Jui | House to house visit | Cluster 1 | N | - | 3 | 0 | C2, the neighbor of C1, had visited C1 secretly for several times during the period of C1 under quarantine at home. C2 developed symptoms on 27th January, but only being detected by local health team via house to house visit until 10th February, which was 13 days later than his onset date. C2 finally recovered and discharged from ETU. |
| C3 | 33  F | Student | Jui | Community report | Cluster 1 | Y | Y | 21 | 3 | C3, who was a nurse student under training and the neighbor of C0, had ever taken care of C0, but she concealed his exposure history and did not being monitored as contact person. C3 developed illness on 1st February, but did not being reported until 7th February. She died on 8th February and then diagnosed as probable Ebola case. |
| C4 | 22  F | Petty trader | Grafton | Community report | Sporadic | N | - | 132 | 0 | C4, a petty trader, frequently travelled to the downtown of Freetown for purchasing goods by taking the mini bus (usually very crowded). C4 was onset on 2nd February, and was reported by her husband via 117 hotline on 9th February. All her 132 contact persons in the family and community (most of them shared the same toilet in the community) were monitored for 21 days, and none of them were infected with ebola. C4 finally recovered and discharged from ETU. Her most likely infectious source is outside of the Grafton community, when travelling to the downtown of Freetown for trading activities. |
| C5 | 10  F | Student | Jui | Contact persons under quarantine | Cluster 1 | Y | Y | 0 | 0 | C5, the daughter of C3, and lived in the same household with C3 when C3 was sick. C5 was registered as the contacts of C3 and had been under quarantined since the C3 was identified as Ebola cases, and then developed symptoms on 14th February. C5 died on 19^th^, February, 2015. |
| C6 | 27  M | Petty trader | Jui | Contact persons under quarantine | Cluster 1 | Y | Y | 0 | 0 | C6, the brother of C3, and took care of C3 when she was sick at home. C6 was registered as the contacts of C3 and had been under quarantined since the C3 was identified as Ebola cases, and then became sick on, 15th, February, 2015. C3 finally died on 18^th^, February, 2015. |
| C7 | 55  F | House wife | Jui | Contact persons under quarantine | Cluster 1 | Y | Y | 0 | 0 | C7, the mother of C3, and took care of C3 when she was sick at home. C7 was registered as the contacts of C3, and had been under quarantine since the C3 was identified as Ebola cases, and then became sick on 18th February. C7 finally died on 22^nd^, February, 2015. |
| C8 | 23  F | NA* | Kossoh Town | Community report | Cluster 2 | Y | Y | 12 | 5 | C8, had ever travelled outside of community before onset on 18th February, and died at home on 25^th^, February. C8 was reported by the neighbor via 117 hotline on 26th February, when her family members planned to hold a traditional burial. The infection sources for C8 is uncertain, but it’s most likely outside of her residence community. |
| C9 | 30  F | Petty trader | Jui | Community report | Sporadic | N | - | 47 | 0 | C9 resided in the downtown of Freetown. On 28th February, she developed sickness, and travelled to visit her sister in Jui community on 2nd March. Her brother-in-law took her to the ETU on 3rd March. All her 47 contact persons were traced, and no secondary case was identified. Her infection source was unidentified, but the most possibility is infected in the Freetown where she residence. C9 finally recovered and discharged from ETU. |
| C10 | 3  M | Child | Kossoh Town | Contact persons under quarantine | Cluster 2 | Y | Y | 0 | 0 | C10, the son of C8, lived with C8 during the period from her illness onset to death, and began to be under quarantine since the date of 26th February. C10 became sick on 5^th^, March, 2015, and finally recovered and discharged from ETU. |
| C11 | 19  M | Welder | Kossoh Town | Contact persons under quarantine | Cluster 2 | N | - | 0 | 0 | C11, the brother of C8, lived with C8 during the period from her illness onset to death, and began to be under quarantine since the date of 26th February. C11 became sick on 5^th^, March, 2015, and finally recovered and discharged from ETU. |
| C12 | 47  F | House wife | Kossoh Town | Contact persons under quarantine | Cluster 2 | Y | Y | 0 | 0 | C12, the mother of C8, lived with C8 during the period from her illness onset to death, and began to be under quarantine since the date of 26^th^ February. C12 became sick on 5^th^, March, 2015, and finally died on 8^th^, March, 2015. |
| C13 | 13  M | Student | Kossoh Town | Contact persons under quarantine | Cluster 2 | N | - | 0 | 0 | C13, the brother of C8, lived with C8 during the period from her illness onset to death, and began to be under quarantine since the date of 26^th^ February. C13 became sick on 12^th^, March, 2015, and finally recovered and discharged from ETU. |
| C14 | 26  F | Student | Kossoh Town | Contact persons under quarantine | Cluster 2 | N | - | 0 | 0 | C12, the sister of C8, lived with C8 during the period from her illness onset to death, and began to be under quarantine since the date of 26^th^ February. C14 became sick on 14^th^, March, 2015, and finally recovered and discharged from ETU. |

* NA：not available.
